# Clinicopathological features, treatment patterns, and survival outcomes among Syrian patients with advanced breast cancer

**DOI:** 10.3389/fonc.2024.1417053

**Published:** 2024-09-12

**Authors:** Muhammad Muhammad, Mousa Alali, Maher Saifo

**Affiliations:** ^1^ Faculty of Medicine, Damascus University, Damascus, Syria; ^2^ Department of Oncology, Albairouni University Hospital, Faculty of Medicine, Damascus University, Damascus, Syria

**Keywords:** advanced breast cancer, prognostic factors, survival, first-line treatment, Syria

## Abstract

**Background:**

Advanced breast cancer (ABC) is a heterogeneous disease with varied prognoses, that is affected by many clinicopathological features. This study aimed to investigate the clinicopathological characteristics, first-line treatment (FLx), and prognostic impact of these features on survival among Syrian patients with ABC.

**Materials and methods:**

This retrospective cohort study included patients with ABC. The association of clinicopathological factors with survival was assessed using Kaplan-Meier curves and the log-rank test, as well as the Cox proportional hazards regression model to calculate the hazard ratio (HaR).

**Results:**

A total of 423 patients with ABC were included in the study, with a median age (range) of 47 years (23-82). 83% of metastases were metachronous. Most patients (91.8%) received chemotherapy as the FLx. The median progression-free survival (PFS) and overall survival (OS) of all the patients were 7 and 16 months, respectively. The median PFS was associated with four factors, which were time of metastasis (adjusted HaR=1.861, 95% CI 1.420-2.438, *P*<0.0001), performance status (PS) (adjusted HaR=1.456, 95% CI 1.049-2.021, *P*=0.025), ovarian metastasis (adjusted HaR=7.907, 95% CI 1.049-59.576, *P*=0.045), and FLx (adjusted HaR=2.536, 95% CI 1.581-4.068, *P*<0.0001). Similarly, the OS was associated with three factors, including hormone receptors (HRs) status (adjusted HaR=1.124, 95% CI 1.009-1.252, *P*=0.034), time of metastasis (adjusted HaR=2.099, 95% CI 1.588-2.775, *P*<0.0001), and PS (adjusted HaR=1.787, 95% CI 1.429-2.233, *P*<0.0001). In the HR-positive/human epidermal growth receptor 2 (HER2)-negative group, endocrine therapy was significantly associated with longer PFS compared with chemotherapy (15 vs 7 months, adjusted HaR=2.699, 95% CI 1.417-5.143, *P*=0.003). Furthermore, there was no difference in OS between the two treatment modalities (*P*=0.855).

**Conclusions:**

ABC survival varies depending on the location of metastases. Good PS and synchronous stage 4 disease were independent prognostic factors for longer PFS and OS. In the HR-positive/HER2-negative group, PFS for endocrine therapy was significantly longer than chemotherapy, with no differences in OS. This study confirms that endocrine therapy is preferred as an FLx for ABC in the HR-positive/HER2-negative group.

## 1 Introduction

Breast cancer (BC) is the second most commonly diagnosed cancer worldwide. BC is the most commonly diagnosed cancer (2,308,897 cases, 23.8%) and the leading cause of cancer-related deaths (665,684 cases, 15.4%) among females in 2022 (1). Similarly, BC is the most common cancer in Syria (24% of all cases in both sexes); Syria has one of the highest mortality rates due to BC among developing countries [Age-Standardized Rate (world), 20.9 per 100,000 females in 2022] (2, 3).

Despite recent advancements in early detection, approximately 10% of patients are diagnosed with advanced breast cancer (ABC) at the initial presentation [*de novo* metastatic breast cancer or synchronous advanced breast cancer (SABC)] (4). Furthermore, in spite of the widespread application of systemic adjuvant therapy, 20 to 30% of patients with BC develop metastatic recurrence following diagnosis and primary treatment, and metastatic cancer contributes to approximately 90% of deaths from BC (5). While BC patients without metastases have a 5-year overall survival (OS) rate of more than 80%, those with distant metastases have an OS rate of only approximately 25% with a median OS of only 2 to 3 years (4). The incidence and mortality of BC vary between developed and developing countries. Various reasons have been attributed to this, including low socioeconomic status, poor diagnostic and treatment practices, insufficient screening programs, and genetic variations (3, 6).

BC tends to metastasize to distinct organs, including the bone, lung, liver, and brain, resulting in varied treatment responses and outcomes. ABC is a heterogeneous disease with distinct prognoses that are influenced by various clinicopathological features of the patient, including age, race, performance status (PS), metastatic sites, number of metastatic sites, and pathological or genotypic characteristics (7). TNM staging is commonly used to predict patient prognosis; however, it addresses only a limited number of factors and does not account for patient-specific conditions, genetic characteristics, and treatments (8). Consequently, an accurate estimation of survival may significantly benefit patients in decision-making.

Numerous studies have investigated the prognosis and outcomes of ABC in developed countries, however, there have been few in the Arab world and the Middle East (9–11). In this study, we investigated the clinicopathological features and first-line treatment (FLx) of Syrian patients with ABC and the prognostic impact of these features on survival.

## 2 Materials and methods

### 2.1 Ethics statement

This study was approved, and a waiver of consent was granted by the Institutional Review Board of the Faculty of Medicine, Damascus University (committee reference number: 2330 on June 13, 2016).

### 2.2 Study design and patient eligibility

This retrospective cohort study was conducted at Albairouni University Hospital (ABUH), a cancer center affiliated with the Syrian Ministry of Higher Education and Scientific Research that treats more than half of all cancer patients in Syria. Due to this, patients come from all Syrian regions.

Inclusion criteria were: age over 18 years, metastatic (clinical stage 4) or locally advanced (refractory to local therapy) BC, between January 1, 2008 and December 31, 2015, with latest follow-up by December 31, 2019. The exclusion criteria included hepatic or renal failure, life-threatening diseases, or cancers other than BC. A total of 423 patients met the inclusion criteria.

### 2.3 Treatment protocol and measurement of clinical factors

Patients considered postmenopausal were those who were: 60 years or older; those with amenorrhea for 12 months or more without prior chemotherapy or ovarian suppression with estradiol and FSH in the postmenopausal range; or those who had amenorrhea as a result of treatment for 12 months with FSH and estradiol in postmenopausal ranges. The PS was assessed using the Eastern Cooperative Oncology Group scale; it was considered good when it was 0-2 and poor when it was 3 and 4. In SABC, the histopathologic, hormone receptors (HRs), and human epidermal growth factor receptor 2 (HER2) status were determined using a biopsy of the metastases or the primary breast cancer; in the case of metachronous advanced breast cancer (MABC), the histopathologic subtype, HRs, and HER2 status were determined using a biopsy of the metastases. BC with <1% staining is considered ER or PR negative. A BC with staining of 1%–100% is considered ER or PR positive; patients with these results are considered eligible for endocrine therapy. Cancers with ER or PR positivity between 1% and 10% are considered low positive, whereas cancers with staining greater than 10% are considered high positive. If either ER or PR or both were positive, BC was considered HR-positive. If both ER and PR were between 1 and 10%, BC was considered HRs-low-positive; If either ER or PR or both were more than 10%, BC was considered HRs-high-positive. HER2 status was determined only by immunohistochemistry [since fluorescence *in-situ* hybridization (FISH) was not available at the ABUH during the study period]; HER2 was considered positive if it was +3 and considered negative otherwise. Regarding grading classification, the Nottingham combined histologic grade (Nottingham modification of the Scarff-Bloom-Richardson grading system) was utilized. In this study, “SABC” (*de novo*) metastases were defined as those occurring within three months of the diagnosis of primary breast cancer; “MABC” (recurrent) metastases occurred three months after the initial diagnosis. Clinical stages were assigned according to the TNM staging (the seventh edition of the American Joint Committee on Cancer) (8).

The primary endpoint was locally assessed progression-free survival (PFS) which was defined as the time from treatment initiation until the occurrence of progression disease (PD) [assessed using the Response Evaluation Criteria in Solid Tumor (RECIST) v1.1] (12), death from any cause, or patient discontinuation from follow-up while receiving treatment. The secondary endpoint was overall survival (OS) which was defined as the time from diagnosis until death from any cause or patient discontinuation from follow-up. Each patient who received one cycle of antitumor treatment was included in the survival assessment.

Treatment was divided into chemotherapy, endocrine therapy (for HR-positive), and anti-HER2-based treatment (for HER2-positive). Treatment was determined according to the opinion of the responsible physician.

### 2.4 Statistical analysis

Using descriptive statistics, data were summarized for numerical variables and presented as mean ± standard deviation (SD) when the data were normally distributed and as median (range) when the data were not normally distributed. Data for nominal variables were summarized using descriptive statistics and are presented as percentages and frequencies.

The Kaplan-Meier method was conducted to estimate survival and was then compared between survival curves by the log-rank test. To determine the prognosis of clinical factors on survival, a univariate Cox proportional hazard analysis was performed with hazard ratios (HaR) and 95% confidence intervals (CI) (*P* value of less than 0.2 by the log-rank test was used to include variables in the univariate Cox model). For factors that were statistically significant in the univariate analysis, a multivariate analysis was conducted. All statistical analyses were conducted using SPSS (version 24). Differences were considered statistically significant if two-sided *P*<0.05.

## 3 Results

### 3.1 Patient characteristics

A total of 423 patients with ABC met the inclusion criteria; their ages ranged from 23 to 82 years at diagnosis, with a median age of 47 years. Most of the patients (58.2%) were premenopausal. The most frequent metastatic sites were the bone (34.8%), liver (23.9%), and lung (21%) and most patients had a single metastatic site (70.4%). Most metastases were metachronous (83%), and most of the patients had good PS (85.1%). **Table 1** shows the demographic characteristics of the study population.

**Table 1:** Baseline demographic characteristics of patients with advanced breast cancer (423 patients).

Characteristics			Total N (%)
**Age (year)**		Median (range)	47 (23-82)
**Menopause status (year)**		Pre menopause	246 (58.2)
		Post menopause	177 (41.8)
**Marital status**		Married	311 (92.8)
		Single	24 (7.2)
		Unknown	88
**Number of children (for married patients)**		0	3 (1)
		1–3	131 (42.1)
		4–6	92 (29.6)
		≥7	82 (27.3)
**Clinical stage**		3 (local, not resectable)	58 (13.7)
		4	365 (86.3)
**Time of metastasis**		Metachronous	351 (83)
		Synchronous	72 (17)
**Metastasis site (single or multiple)**	Bone		147 (34.8)
	Liver		101 (23.9)
	Lung		89 (21)
	Local		83 (19.6)
	Brain		17 (4)
	Other	Lymph node	37 (8.7)
		Skin	18 (4.3)
		Pleural effusion	42 (9.9)
		Another breast	13 (3.1)
		Ascites	6 (1.4)
		Pericardial effusion	1 (0.2)
		Ovary	1 (0.2)
		Spleen	1 (0.2)
**Number of metastasis site**		1	298 (70.4)
		2	92 (21.7)
		3	29 (6.9)
		4	4 (0.9)
**Number of metastasis site**		2 or less	390 (92.2)
		3 or more	33 (7.8)
**CEA status**		Normal	157 (74.4)
		Increased	54 (25.6)
		Unknown	212
**CA15-3 status**		Normal	134 (54)
		Increased	114 (46)
		Unknown	175
**ECOG performance status**	Good 348 (85.1)	0	80 (19.6)
		1	141 (34.5)
		2	127 (31.1)
	Poor 61 (14.9)	3	46 (11.2)
		4	15 (3.7)
	Unknown		14

CA15-3, cancer antigen 15-3; CEA, carcinoembryonic antigen; ECOG, Eastern Cooperative Oncology Group.

Invasive ductal carcinoma (IDC) was the most common histological subtype (89.8%). The HRs status was positive in 62%, HER2 was positive in 31.3%, HR-positive/HER2-negative was 40.5%, and triple-negative BC (TNBC) was 23.4%. Grade 2 was the most common histological grade (50.6%) (**Table 2**). Most patients received chemotherapy as FLx (91.8%) (**Table 3**).

**Table 2:** Pathological characteristics of patients with advanced breast cancer (423 patients).

Characteristics				Total N (%)
**Histology**			IDC	362 (89.8)
			ILC	39 (9.7)
			PC	2 (0.5)
			Unknown	20
**Grade**			G1	10 (2.9)
			G2	175 (50.6)
			G3	161 (46.5)
			Unknown	77
**Hormone receptors and HER2 status**	ER		Positive	184 (45)
			Negative	225 (55)
			Unknown	14
	PR		Positive	221 (54)
			Negative	188 (46)
			Unknown	14
	HR		Positive	255 (62)
			Negative	156 (38)
			Unknown	12
	HER2	Negative 263 (68.7)	0	131 (34.2)
			+1	55 (14.4)
			+2	77 (20.1)
		Positive 120 (31.3)	+3	120 (31.3)
		Unknown		40
**Molecular subtype**			HR-positive/HER2-negative	166 (40.5)
			HR-positive/HER2-positive	68 (16.6)
			HR-negative/HER2-positive	52 (12.7)
			TNBC	96 (23.4)
			HR-positive/unknown	21 (5.1)
			HR-negative/unknown	7 (1.7)
			Unknown	13

ER, estrogen receptor; IDC, invasive ductal carcinoma; ILC, invasive lobular carcinoma; HER2, human epidermal growth factor receptor 2; HR, hormone receptor; PC, papillary carcinoma; PR, progesterone receptor; TNBC, triple-negative breast cancer.

**Table 3:** First-line treatment of patients with advanced breast cancer (423 patients).

First-line treatment			Total N (%)
Chemotherapy, 345 (91.8)	Anthracycline based**, 49 (13)	AC	15 (4)
		FAC	34 (9)
	TXN based***, 115 (30.6)	TXN	64 (17)
		Platinum**** + TXN	33 (8.8)
		TC	9 (2.4)
		GMZ + TXN	9 (2.4)
	Anthracycline/TXN based	AT	35 (9.3)
	Other, 146 (38.9)	Capecitabine	14 (3.7)
		CMF	8 (2.1)
		GMZ+NVB	1 (0.3)
		Platinum + GMZ	4 (1.1)
		Platinum + NVB	87 (23.1)
		NVB	15 (4)
		NVB + capecitabine	17 (4.5)
Endocrine therapy*			26 (6.9)
Anti-HER2-based therapy, 5 (1.3)		GMZ + TXN + trastuzumab	1 (0.3)
		Platinum + NVB + trastuzumab	2 (0.5)
		TPH	2 (0.5)
Unknown			47

*Includes aromatase inhibitors, tamoxifen, and ovarian suppression. **Includes doxorubicin or epirubicin. ***Includes docetaxel or paclitaxel. ****includes carboplatin or cisplatin.

AC, doxorubicin and cyclophosphamide; AT, doxorubicin and docetaxel; CMF, cyclophosphamide, methotrexate, and fluorouracil; FAC, fluorouracil, doxorubicin and cyclophosphamide; GMZ, gemcitabine; HER2, human epidermal growth factor receptor 2; NVB, vinorelbine; TC, docetaxel and cyclophosphamide; TPH, taxane, platin, and trastuzumab; TXN, taxane.

### 3.2 Survival outcomes and variables affecting the outcomes

#### 3.2.1 The overall population

##### 3.2.1.1 PFS and its related factors

Median follow-up from ABC diagnosis was 107 months (8.9 years). The median PFS of all the patients was 7 months (95% CI 6.45-7.54) (**Supplementary Figure S1**). Other than TNBC molecular subtypes, SABC, good PS, endocrine therapy, IDC histopathologic subtype (**Figures 1A–E**), and bone metastasis (**Figure 2B** and **Supplementary Figure S14**), were associated with longer PFS according to the log-rank test. In contrast, brain metastasis and ovarian metastasis were associated with shorter PFS according to the log-rank test (**Figures 2A, C**, respectively). Other laboratory or clinical factors were not associated with PFS (**Supplementary Figures S2**-**S13**, **S15**-**S28**).

**Figure 1:**
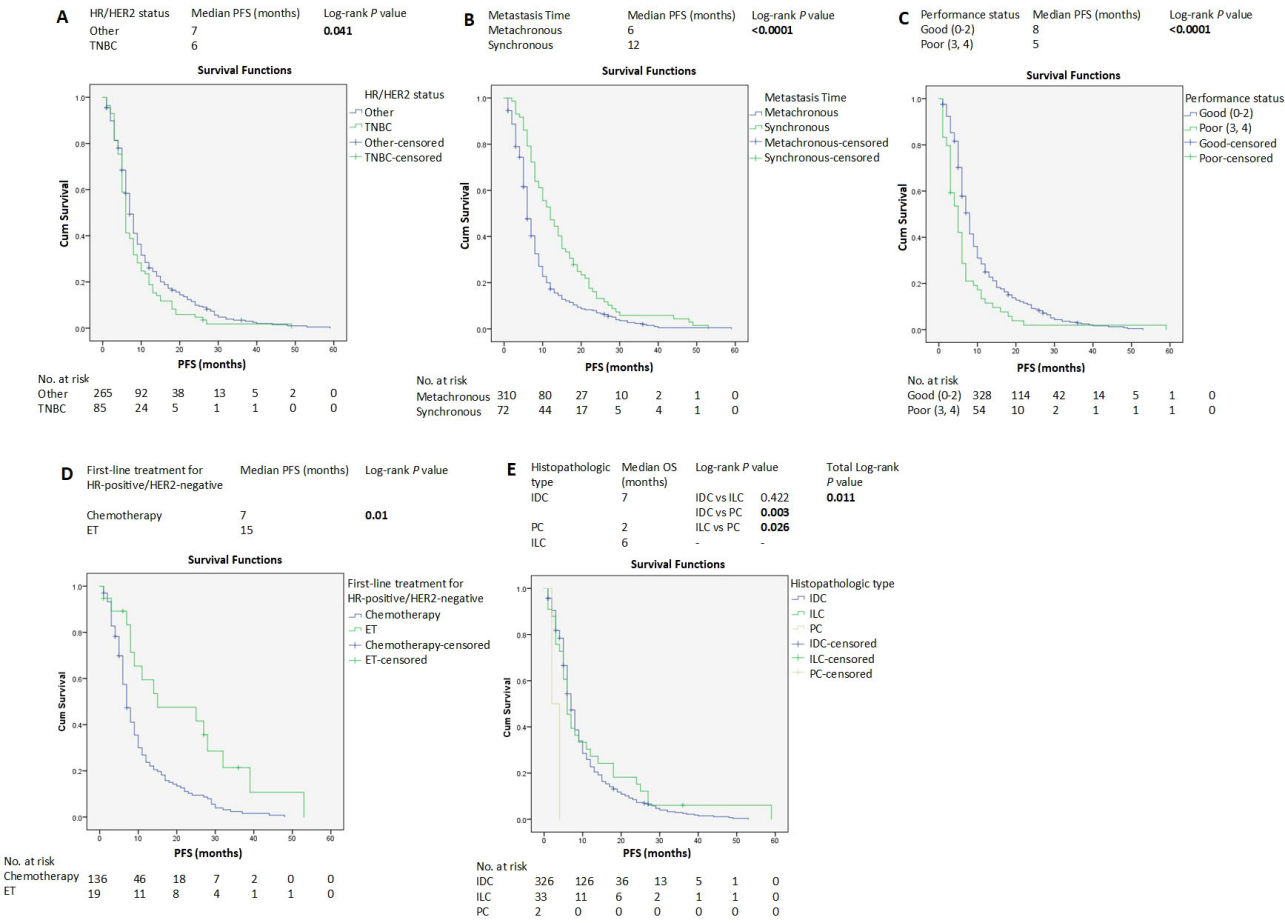
Kaplan–Meier estimates of PFS of patients with advanced breast cancer according to clinicopathological factors and treatments **(A-E)**. HER2, human epidermal growth factor receptor 2; HR, hormone receptors; ET, endocrine therapy; IDC, invasive ductal carcinoma; ILC, invasive lobular carcinoma; PC, papillary carcinoma; PFS, progression-free survival.

**Figure 2:**
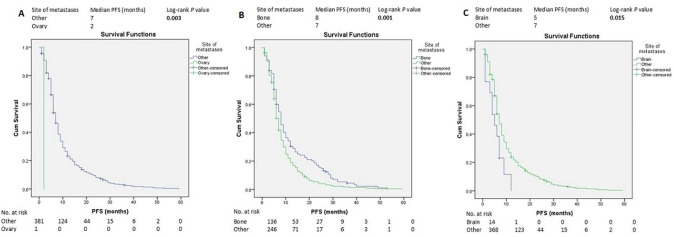
Kaplan–Meier estimates of PFS of patients with advanced breast cancer according to the metastasis site **(A-C)**. PFS, progression-free survival.

There was no difference in PFS when comparing HR-low-positive and HR-high-positive (**Supplementary Figure S7**). Similarly, there was no difference in PFS when comparing HER2 + 1 and HER2 + 2 (**Supplementary Figure S9**).

For FLx, PFS was 19 months for endocrine therapy vs 7 months for chemotherapy-based therapy (*P*<0.0001) (**Supplementary Figures S29**, **S30**).

Univariate and multivariate Cox regression models showed that time of metastasis, PS, FLx, and ovarian metastasis were independent factors associated with PFS (**Table 4**).

**Table 4:** Univariate and multivariate analyses of clinicopathological factors associated with PFS of patients with advanced breast cancer (423 patients).

Variable			Uni-variate Cox regression PFS			Multi-variate Cox regression PFS		
			HaR	95% CI	*P* value	HaR	95% CI	*P* value
**Number of metastases**		>2	1.00 (ref)	–	–	–	–	–
		≤2	0.888	0.736-1.071	0.215	–	–	–
**Presence of bone metastasis**		No	1.00 (ref)	–	–	1.00 (ref)	–	–
		Yes	1.392	1.120-1.730	**0.003**	1.131	0.900-1.421	0.291
**Presence of brain metastasis**		Yes	1.00 (ref)	–	–	1.00 (ref)	–	–
		No	1.394	1.043-1.862	**0.025**	1.341	0.728-2.469	0.347
**Presence of ovary metastasis**		Yes	1.00 (ref)	–	–	1.00 (ref)	–	–
		No	10.592	1.451-77.318	**0.020**	7.907	1.049-59.576	**0.045**
**Presence of liver metastasis**		Yes	1.00 (ref)	–	–	–	–	–
		No	1.092	0.967-1.234	0.156	–	–	–
**Presence of pericardial metastasis**		Yes	1.00 (ref)	–	–	–	–	–
		No	4.132	0.575-29.675	0.158	–	–	–
**HR/HER2 status**		TNBC	1.00 (ref)	–	–	–	–	–
		Other	1.276	0.995-1.637	0.055	–	–	–
**Time of metastasis**		Meta	1.00 (ref)	–	–	1.00 (ref)	–	–
		Syn	1.750	1.345-2.277	**<0.0001**	1.861	1.420-2.438	**<0.0001**
**ECOG PS**		Poor (3, 4)	1.00 (ref)	–	–	1.00 (ref)	–	–
		Good (0-2)	1.672	1.24-2.249	**0.001**	1.456	1.049-2.021	**0.025**
**First-line treatment**		Other	1.00 (ref)	–	–	1.00 (ref)	–	–
		ET	2.506	1.58-3.95	**<0.0001**	2.536	1.581-4.068	**<0.0001**

CI, confidence interval; ECOG, Eastern Cooperative Oncology Group; ET, endocrine therapy; HaR, hazard ratio; HR, hormone receptors; HER2, human epidermal growth factor receptor 2; PFS, progression-free survival; PS, performance status; Meta, metachronous; Syn, synchronous.

The bold values indicate statistical significance (*P*<0.05).

##### 3.2.1.2 OS and its related factors

The median OS of all the patients was 16 months (95% CI 14.35-17.64), and the Kaplan–Meier estimate of OS at 60 months was 4.1% (**Supplementary Figure S31**).

**Figure 3:**
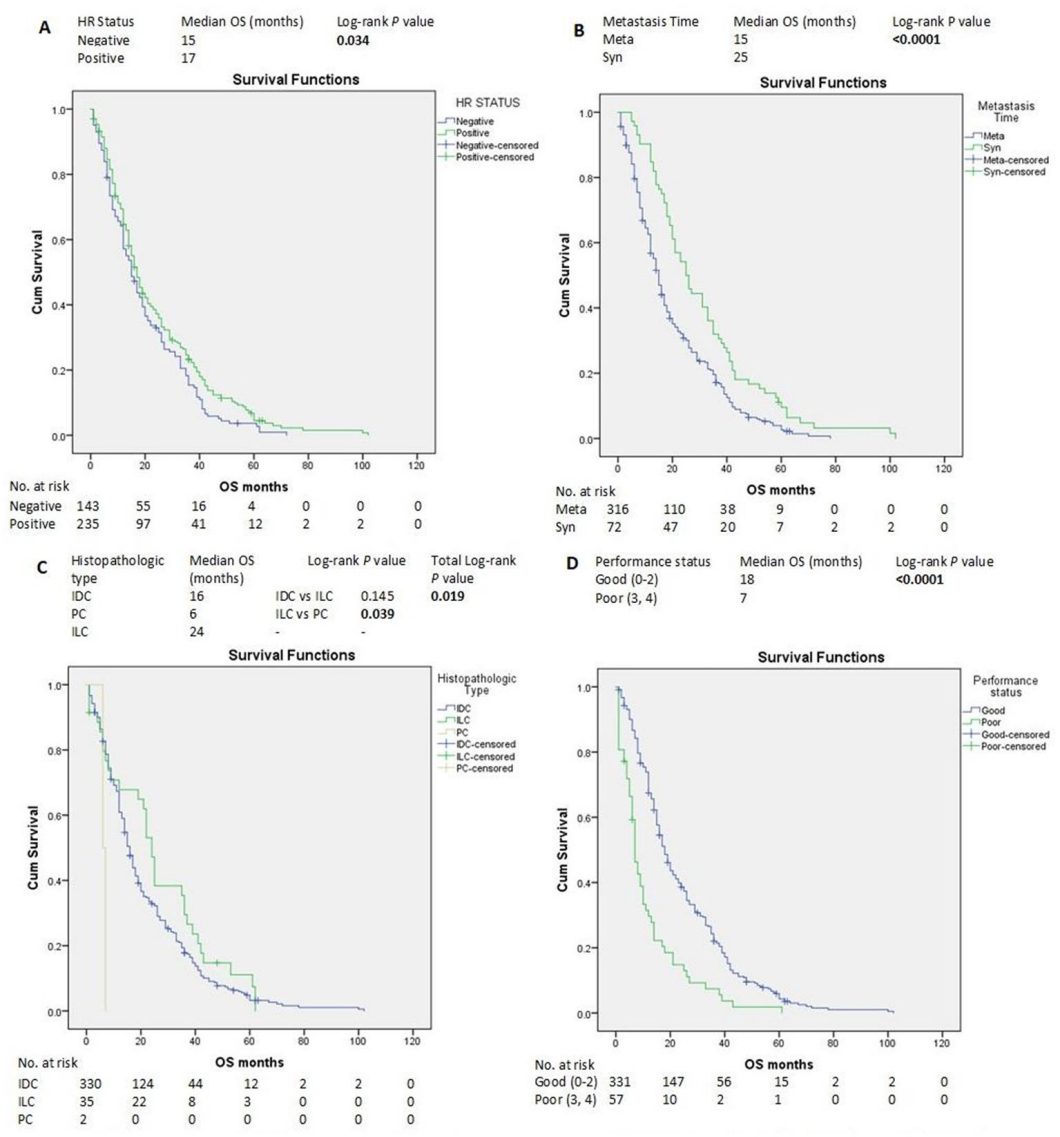
Kaplan–Meier estimates of OS of patients with advanced breast cancer according to clinicopathological factors and treatments **(A-D)**. HR, hormone receptors; IDC, invasive ductal carcinoma; ILC, invasive lobular carcinoma; Meta, metachronous; OS, overall survival; PC, papillary carcinoma; Syn, synchronous.

**Figure 4:**
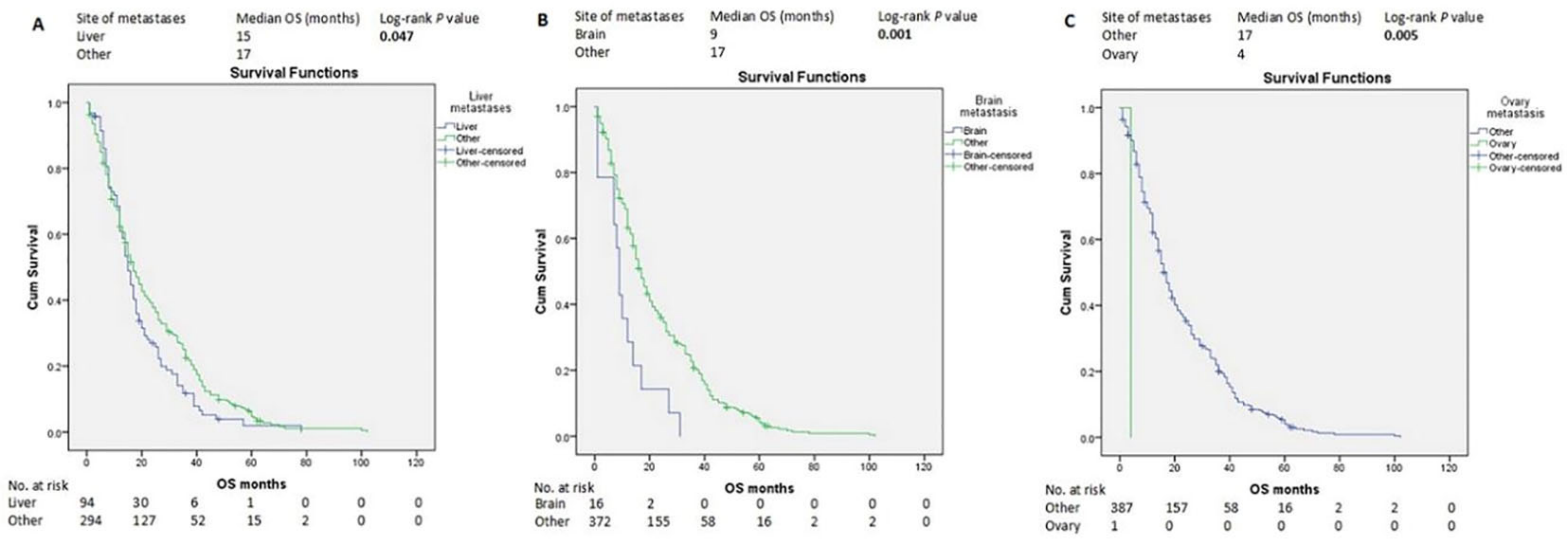
Kaplan–Meier estimates of OS of patients with advanced breast cancer according to the metastasis site **(A-C)**. OS, overall survival.

HR-positive status, SABC, good PS, and ILC histopathological subtype were associated with longer OS according to the log-rank test (**Figures 3A–D**). In contrast, liver, brain, and ovarian metastases were associated with shorter OS (**Figures 4A–C**).

OS in patients with PR-low-positive was longer than with PR-negative (*P*=0.03), however, there was no difference in OS when comparing PR-low-positive and PR-high-positive (*P*=0.137) or between HRs expression levels (*P*=0.091) (**Supplementary Figures S35**-**S37**). There were no differences in the OS between HER2 + 1 and HER2 + 2 (**Supplementary Figure S38**). TNBC and G3 were associated with worse survival than HR-positive/HER2-negative and G1, respectively (*P*=0.040 and *P*=0.01, respectively) (**Supplementary Figures S40**-**S42**).

For FLx, OS was 25 months for endocrine therapy vs 26 months for anthracycline-based chemotherapy, 18 months for taxane-based chemotherapy, and 14 months for other chemotherapies, and log-rank test when comparing endocrine therapy vs chemotherapy was insignificant (**Supplementary Figures S58**, **S60**). Other laboratory or clinical factors were not associated with OS (**Supplementary Figures S32**-**S34**, **S38**, **S39**, **S43**-**S57**).

Univariate and multivariate Cox regression models showed that the HRs status, time of metastasis, and PS were independent factors associated with OS (**Table 5**).

**Table 5:** Univariate and multivariate analyses of clinicopathological factors associated with OS of patients with advanced breast cancer (423 patients).

Variable			Uni-variate Cox regression OS			Multi-variate Cox regression OS		
			HaR	95% CI	*P* value	HaR	95% CI	*P* value
**Histopathologic type**		IDC	1.00 (ref)	–	–	–	–	–
		ILC	1.297	0.905-1.860	0.157	–	–	–
**HR**		Negative	1.00 (ref)	–	–	1.00 (ref)	–	–
		Positive	1.119	1.006-1.246	**0.039**	1.124	1.009-1.252	**0.034**
**Presence of brain metastasis**		Yes	1.00 (ref)	–	–	1.00 (ref)	–	–
		No	1.538	1.174-2.014	**0.002**	1.255	0.951-1.657	0.108
**Presence of ovary metastasis**		Yes	1.00 (ref)	–	–	1.00 (ref)	–	–
		No	9.771	1.342-71.168	**0.024**	5.871	0.799-43.127	0.082
**Presence of liver metastasis**		Yes	1.00 (ref)	–	–	–	–	–
		No	1.127	0.998-1.272	0.053	–	–	–
**Time of metastasis**		Meta	1.00 (ref)	–	–	1.00 (ref)	–	–
		Syn	1.299	1.138-1.484	**<0.0001**	2.099	1.588-2.775	**<0.0001**
**ECOG PS**		Poor (3, 4)	1.00 (ref)	–	–	1.00 (ref)	–	–
		Good (0-2)	2.331	1.743-3.117	**<0.0001**	1.787	1.429-2.233	**<0.0001**

CI, confidence interval; ECOG, Eastern Cooperative Oncology Group; HaR, hazard ratio; HR, hormone receptors; OS, overall survival; PS, performance status; Meta, metachronous; Syn, synchronous.

The bold values indicate statistical significance (*P*<0.05).

#### 3.2.2 The HR-positive/HER2-negative group

##### 3.2.2.1 PFS and its related factors

The median PFS of the HR-positive/HER2-negative group was 8 months (95% CI 7.025-8.975) (**Supplementary Figure S11**). For FLx, median PFS was significantly longer in the endocrine therapy group compared with the chemotherapy-based group (15 vs 7 months, *P*=0.01) (**Figure 1D**).

Univariate and multivariate Cox regression models showed that time of metastasis (adjusted HaR=2.302, 95% CI 1.488-3.560, *P*<0.0001), PS (adjusted HaR=2.056, 95% CI 1.771-3.609, *P*=0.012), and FLx (adjusted HaR=2.699, 95% CI 1.417-5.143, *P*=0.003), were independent factors associated with PFS (**Table 6**).

**Table 6:** Univariate and multivariate analyses of clinicopathological factors associated with PFS of patients with HR-positive/HER2-negative (166 patients).

Variable			Uni-variate Cox regression PFS			Multi-variate Cox regression PFS		
			HaR	95% CI	*P* value	HaR	95% CI	*P* value
**Number of metastases**		>2	1.00 (ref)	–	–	–	–	–
		≤2	0.882	0.674-1.154	0.360	–	–	–
**Presence of bone metastasis**		No	1.00 (ref)	–	–	1.00 (ref)	–	–
		Yes	1.487	1.005-2.200	**0.047**	1.006	0.640-1.583	0.979
**Presence of brain metastasis**		Yes	1.00 (ref)	–	–	–	–	–
		No	1.064	0.599-1.889	0.833	–	–	–
**Presence of liver metastasis**		Yes	1.00 (ref)	–	–	–	–	–
		No	1.090	0.877-1.355	0.435	–	–	–
**Presence of pericardial metastasis**		Yes	1.00 (ref)	–	–	–	–	–
		No	4.542	0.620-33.277	0.136	–	–	–
**Time of metastasis**		Meta	1.00 (ref)	–	–	1.00 (ref)	–	–
		Syn	1.359	1.102-1.676	**0.004**	2.302	1.488-3.560	**<0.0001**
**ECOG PS**		Poor (3, 4)	1.00 (ref)	–	–	1.00 (ref)	–	–
		Good (0-2)	1.802	1.049-3.093	**0.033**	2.056	1.771-3.609	**0.012**
**First-line treatment**		Other	1.00 (ref)	–	–	1.00 (ref)	–	–
		ET	1.554	1.14-2.056	**0.002**	2.699	1.417-5.143	**0.003**

CI, confidence interval; ECOG, Eastern Cooperative Oncology Group; ET, endocrine therapy; HaR, hazard ratio; HR, hormone receptors; HER2, human epidermal growth factor receptor 2; PFS, progression-free survival; PS, performance status; Meta, metachronous; Syn, synchronous.

The bold values indicate statistical significance (*P*<0.05).

##### 3.2.2.2 OS and its related factors

The median OS of the HR-positive/HER2-negative group was 18 months (95% CI 14.491-21.509), and the Kaplan–Meier estimate of OS at 60 months was 5.1% (**Supplementary Figure S40**). Although OS on endocrine therapy was 27 months compared to 17 months for chemotherapy as FLx, there was no difference in the OS between the two groups (*P*=0.855). (**Supplementary Figure S59**).

Univariate and multivariate Cox regression models showed that the time of metastasis (adjusted HaR=1.451, 95% CI 1.172-1.797, *P*=0.001), and PS (adjusted HaR=2.553, 95% CI 1.493-4.365, *P*=0.001) were independent factors associated with OS (**Table 7**).

**Table 7:** Univariate and multivariate analyses of clinicopathological factors associated with OS of patients with HR-positive/HER2-negative (166 patients).

Variable			Uni-variate Cox regression OS			Multi-variate Cox regression OS		
			HaR	95% CI	*P* value	HaR	95% CI	*P* value
**Histopathologic type**		IDC	1.00 (ref)	–	–	–	–	–
		ILC	1.256	0.732-2.156	0.408	–	–	–
**Presence of brain metastasis**		Yes	1.00 (ref)	–	–	–	–	–
		No	1.643	0.989-2.730	0.055	–	–	–
**Presence of liver metastasis**		Yes	1.00 (ref)	–	–	–	–	–
		No	1.097	0.889-1.353	0.389	–	–	–
**Time of metastasis**		Meta	1.00 (ref)	–	–	1.00 (ref)	–	–
		Syn	1.380	1.118-1.703	**0.003**	1.451	1.172-1.797	**0.001**
**ECOG PS**		Poor (3, 4)	1.00 (ref)	–	–	1.00 (ref)	–	–
		Good (0-2)	2.094	1.239-3.541	**0.006**	2.553	1.493-4.365	**0.001**

CI, confidence interval; ECOG, Eastern Cooperative Oncology Group; HaR, hazard ratio; HER2, human epidermal growth factor receptor 2; HR, hormone receptors; OS, overall survival; PS, performance status; Meta, metachronous; Syn, synchronous.

The bold values indicate statistical significance (*P*<0.05).

## 4 Discussion

Despite all available treatments, ABC remains a global problem owing to its high incidence, aggressiveness, and fatality rate. ABC is an incurable disease that usually results in death with a 5-year survival rate of less than 25% (4, 5). The survival of patients with ABC varies among individuals and between developing and developed countries (3, 6). Hence, it is important to determine the prognosis and predict outcomes. This study was conducted in a developing country (Syria) and identified the most important prognostic factors and FLx and it showed that several clinicopathologic factors impacted survival, while other factors had no effect.

In our study, approximately 60% of patients were pre-menopausal. In a review of Arab countries, it was reported that the median age at diagnosis in the Arab population was 48 years old and that two-thirds of BC patients were younger than 50 years old (13). This study highlights the early onset of BC among Syrians compared to Western populations. Among the reasons might be a younger population structure, different environmental factors, as well as differences in screening practices and genetic makeup (14).

The most aggressive BC, TNBC, does not express ER, PR, or HER2. It is usually detected at an advanced stage when diagnosed, resulting in a high recurrence rate and poor survival. TNBC accounts for approximately 15 to 20% of BC cases (15). Several drugs targeting TNBC [such as Immunotherapy, poly (ADP-ribose) polymerase inhibitors (PARPIs), and antibody-drug conjugates] have been approved by the US FDA in recent years, which have contributed to improving the prognosis of patients with metastatic TNBC (16, 17). TNBC constituted nearly a quarter of the cases (23.4%) in our study, which is a relatively high percentage compared to neighboring countries such as Iraq (7.2%) and Kuwait (12%) (18, 19). In the current study, there were approximately 40% of patients with HR-positive/HER2-negative and more than 50% with HER2-positive and TNBC combined. Molecular subtype status reflects poor prognosis in the study population. The percentage is similar to that reported in the UAE, where ER-positive, PR-positive, HER2-positive, and TNBC tumor incidence was 59.3%, 51.0%, 39.1%, and 20.8%, respectively (20). In addition to ethnic factors, this may also be due to the fact that ABCs were included in the sample. Based on a study that examined the differences in biological features between primary and recurrent tumors, ER positivity decreased from 61.4% to 58.6% and PR positivity decreased from 61.4% to 44.3%, with an increase in HER2 positivity and overall changes were seen in 5.7% of cases (21).

The median OS in our study was 16 months, whereas in previous studies, it ranged from 18 to 63.9 months (22–27). The survival duration in our study was shorter than that reported in the previous studies. This may be due to the different inclusion criteria. This is a real-world study in which patients were included regardless of PS. In contrast, some previous studies were limited to fit patients (PS = 0, 1) (27). Furthermore, in our study, most patients were administered chemotherapy or endocrine monotherapy (because targeted agents were not available until the end of follow-up), which may explain the longer OS in previous studies compared with ours. Finally, our study was conducted during the Syrian crisis, which may have caused leakage or death in some patients.

This study showed that MABC had a poor prognosis compared to SABC (*P*<0.0001 for PFS and OS). Several studies have also reported similar results (22, 26, 28). Prior lines of systemic treatment after primary breast cancer treatment could modify the course of the metachronic disease. Therefore, the SABC and MABC may represent distinct entities with respect to their biological behavior. Optimal clinical management may require different strategies for synchronous and metachronous metastases. The majority of ABC cases were MABC (in our study, 83%), which confirms the necessity of early detection of BC along with providing appropriate adjuvant treatments to reduce recurrence rate and improve the prognosis of ABC.

The metastatic potential of BC includes the bone, lung, liver, brain, and others. Previous studies have reported conflicting prognoses regarding the location of metastases. Most of these studies showed that the prognosis of bone metastases was better than that of visceral metastases (24, 29–31). Some studies showed that brain metastases were worse than other visceral metastases (29, 30), whereas others found pleural metastases was associated with poor outcomes (32). Other studies have indicated that ovarian metastasis to be worse (33). In this study, multivariate analysis demonstrated that ovarian metastasis was associated with shorter PFS than other metastases. However, the location of metastases did not affect OS. Several previous studies have shown that multiple tumor metastases was poor prognostic factor (24, 29). However, others have not shown a significant effect on survival. There may be an effect of the location or an effect of the large tumor burden of the metastases rather than their number.

PS is an important factor in predicting PFS and OS (24), particularly in developing countries because many patients are diagnosed late with poor PS due to economic, social, or even security reasons (as in Syria’s case) (34).

Despite recent medical advances, metastasis remains the most common cause of death in patients with BC. The mechanisms that lead to BC metastasis have been intensively studied, and drugs have been developed to inhibit these mechanisms. However, it cannot prevent the death of patients from metastasis, because metastasis is not triggered by a single factor but by several factors (17).

Recent guidelines have reported that a combination of aromatase inhibitor and cyclin4/6-dependent kinase inhibitors (CDKIs) is the preferred FLx for HR-positive/HER2-negative BC (35). The MONALEESA-2 study (ClinicalTrials.gov Identifier, NCT01958021) demonstrated an improvement in PFS (25.3 vs 16.0 months; HaR=0.56) and OS (63.9 vs 51.4 months; HaR=0.76) was seen with ribociclib plus letrozole compared with letrozole alone (27). Several previous studies have concluded that endocrine monotherapy has better outcomes than chemotherapy in HR-positive/HER2-negative group. In the study by Jacquet et al. (23), the median PFS was 15.1 months in the endocrine therapy group compared with 12.5 months in the chemotherapy group (*P*<0.0001), while the median OS was 60.7 months in the endocrine therapy group compared with 49.6 months in the chemotherapy group (*P*<0.0001). In the current study, a significant increase in PFS was found with endocrine therapy as FLx for HR-positive/HER2-negative ABC compared to chemotherapy (15 vs 7 months, *P*=0.01), which is consistent with previous studies (23, 36). Even though endocrine therapy is preferable over PFS and quality of life (in the absence of a visceral crisis), and approximately 40% of BC in our study were HR-positive/HER2-negative, only 6.9% of patients received endocrine therapy. The reason is that oncologists don’t adhere to the guidelines, which is common even in developed countries, since previous studies showed that more than 50% of ABCs were treated with chemotherapy as FLx (23, 24, 35, 36). In addition, this study indicated that the results of endocrine therapy are comparable to those of chemotherapy in terms of OS. This indicates the importance of increasing oncologists’ awareness of the superiority of endocrine therapy over chemotherapy in HR-positive/HER2-negative BC and the necessity of adhering to international guidelines. Anthracycline- and taxane-based chemotherapy showed better results in this study than the other chemotherapy protocols. Each patient requires a tailored approach, with the treatment option based on their general health status and the presence of a visceral crisis being assessed.

A systematic and in-depth study of the molecular heterogeneity of metastatic BC could therefore result in the discovery of more effective agents for treating metastasis and improved prognosis for patients by identifying the possible causes of many therapeutic failures. Further research is required to validate the identified genes and molecular mechanisms for future clinical applications. The anti-PD-L1 therapeutic antibodies have demonstrated significant antitumor activity in ABC, suggesting that immune checkpoints have a significant impact on BC metastatic cascades; No single drug treatment can permanently eliminate the tumor, according to many studies (37). The combination of immune checkpoint inhibitors and existing treatments for ABC is a promising strategy, that requires extensive testing in clinical trials (38).

Our study has some limitations. First, this study did not use the FISH because it is not available at the time of diagnosis in ABUH, so HER2 + 2 (equivocal) by immunohistochemistry was considered HER2-negative. HER2 + 2 BC is a heterogeneous group, however, in most cases, it was reclassified as HER2-negative (39, 40). Furthermore, PD-L1, BRCA, and other genetic tests were not performed in this study, which were not available in Syrian public hospitals until recently. Most patients were not tested for KI-67, therefore, the molecular subtypes were identified based only on HRs and HER2 status. In addition, most patients did not receive anti-HER2 therapy (for HER2-positive), nor did they receive CDKIs or PARPIs, which may have contributed to poor survival. Currently, several anti-HER2 agents are available at ABUH (trastuzumab, pertuzumab, and trastuzumab emtansine), however, these agents are frequently interrupted, and several targeted agents used to treat BC or other cancers do not exist because of a lack of resources (41). Finally, since the study was retrospective, some patients’ information was missing, such as occupation, and educational level, so these factors were not examined.

In conclusion, ABC survival varies according to the location of metastases, especially in the bone, brain, liver, and ovary. There were no differences in survival between patients with PR-low-positive and PR-high-positive or HER2 + 1 and HER2 + 2. Synchronous stage 4 disease and good PS were independent prognostic factors for predicting longer PFS and OS in patients with ABC. Most patients with ABC still receive chemotherapy as an FLx in the HR-positive/HER2-negative group in Syria. Endocrine therapy was associated with better PFS than chemotherapy, with no differences in OS. This study confirms the use of endocrine therapy as an FLx for the treatment of HR-positive/HER2-negative ABC, especially when there are no cases requiring the use of chemotherapy.

## Data Availability

The raw data supporting the conclusions of this article will be made available by the authors, without undue reservation.
