# Supplementary material for: Clinicopathological features, treatment patterns, and survival outcomes among Syrian patients with advanced breast cancer
Source: Front Oncol. 2024 Sep 12;14:1417053. doi: 10.3389/fonc.2024.1417053 (PMC11424526; doi:10.3389/fonc.2024.1417053)
Supplement: Supplementary file 1 [file DataSheet1.docx]

**Supplementary material contents:**

1. **Progression-free survival (PFS) analysis according to clinicopathological features for patients with advanced breast cancer (423 patients)** **(Supplementary Figures S1-S30)**
2. **Overall survival (OS) analysis according to clinicopathological features for patients with advanced breast cancer (423 patients) (Supplementary Figures S31-S60)**
3. **Progression-free survival (PFS) analysis according to clinicopathological features for patients with advanced breast cancer (423 patients)**

| Mean ± SD PFS (months) | Median PFS (months) | Range (months) | 95% CI |
| --- | --- | --- | --- |
| 9.73 ± 8.862 | 7 | 1-59 | 6.45-7.54 |


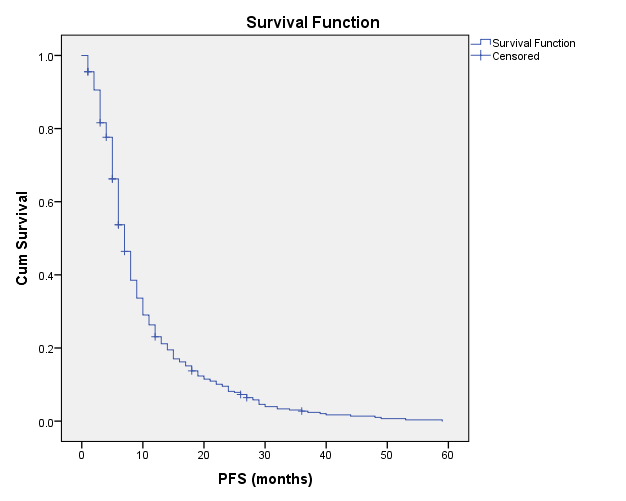


| Months | 0 | 10 | 20 | 30 | 40 | 50 | 60 |
| --- | --- | --- | --- | --- | --- | --- | --- |
| No. at risk | 382 | 124 | 44 | 15 | 6 | 2 | 0 |

**Supplementary Figure S1. Kaplan–Meier estimate of PFS of patients with advanced breast cancer.**

CI, confidence interval; PFS, progression-free survival; SD, standard deviation.

| Menopause status | Median PFS (months) | Log-rank *P* value |
| --- | --- | --- |
| Post | 7 | 0.645 |
| Pre | 7 |  |


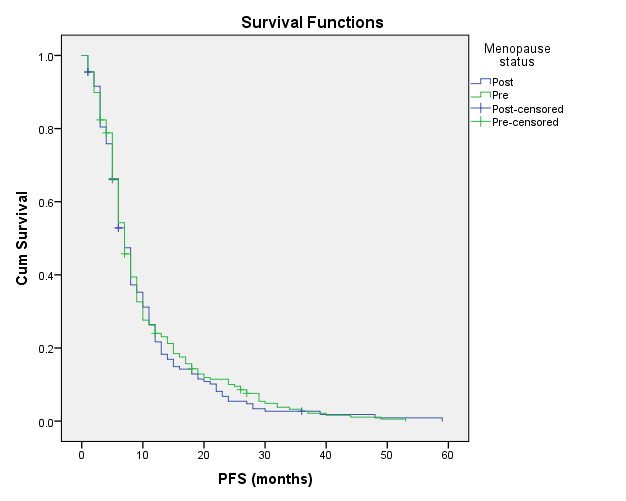


| No. at risk | | | | | | | |
| --- | --- | --- | --- | --- | --- | --- | --- |
| Months | 0 | 10 | 20 | 30 | 40 | 50 | 60 |
| Post | 155 | 52 | 17 | 5 | 2 | 1 | 0 |
| Pre | 227 | 72 | 27 | 10 | 4 | 1 | 0 |

**Supplementary Figure S2. Kaplan–Meier estimates of PFS according to menopause status.**

PFS, progression-free survival.

| ER Status | Median PFS (months) | Log-rank *P* value |
| --- | --- | --- |
| Negative | 6 | 0.357 |
| Positive | 7 |  |


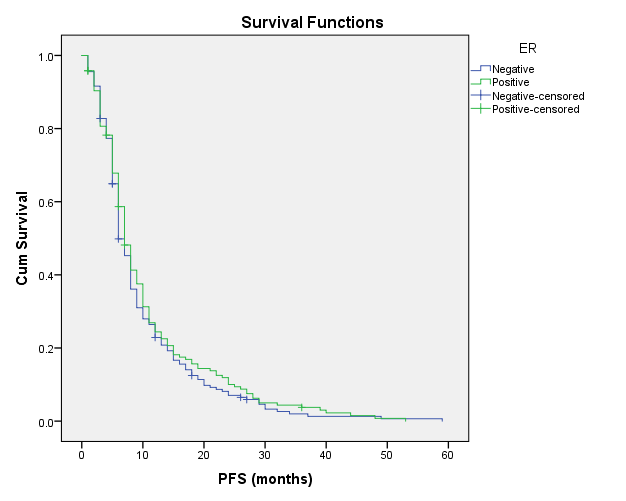


| No. at risk | | | | | | | |
| --- | --- | --- | --- | --- | --- | --- | --- |
| Months | 0 | 10 | 20 | 30 | 40 | 50 | 60 |
| Negative | 204 | 61 | 21 | 7 | 2 | 1 | 0 |
| Positive | 166 | 60 | 23 | 8 | 4 | 1 | 0 |

**Supplementary Figure S3. Kaplan–Meier estimates of PFS according to ER status.**

ER, estrogen receptor; PFS, progression-free survival.

| ER Status | Median PFS (months) |  | Log-rank *P* value | Log-rank *P* value (overall) |
| --- | --- | --- | --- | --- |
| Negative | 6 | Negative vs High | 0.453 | 0.631 |
| Low | 7 | Low vs High | 0.846 |  |
| High | 7 | - |  |  |


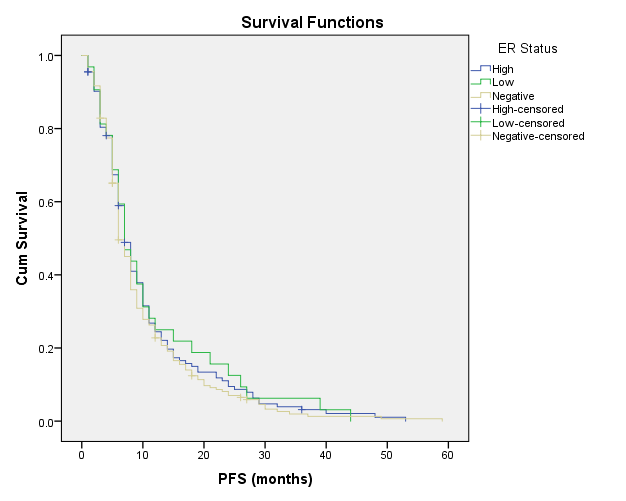


| No. at risk | | | | | | | |
| --- | --- | --- | --- | --- | --- | --- | --- |
| Months | 0 | 10 | 20 | 30 | 40 | 50 | 60 |
| Negative | 204 | 61 | 21 | 7 | 2 | 0 | 0 |
| Low | 32 | 12 | 6 | 2 | 1 | 0 | 0 |
| High | 134 | 48 | 17 | 6 | 3 | 1 | 0 |

**Supplementary Figure S4. Kaplan–Meier estimates of PFS according to ER status (three categories).**

ER, estrogen receptor; PFS, progression-free survival.

| PR Status | Median PFS (months) | Log-rank *P* value |
| --- | --- | --- |
| Negative | 7 | 0.084 |
| Positive | 7 |  |


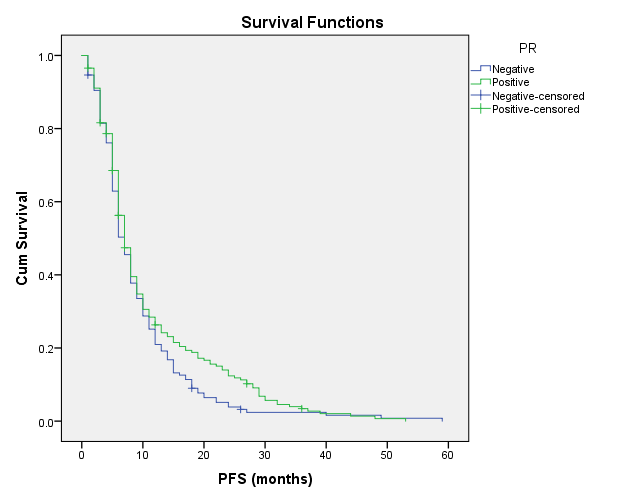


| No. at risk | | | | | | | |
| --- | --- | --- | --- | --- | --- | --- | --- |
| Months | 0 | 10 | 20 | 30 | 40 | 50 | 60 |
| Negative | 168 | 56 | 12 | 3 | 3 | 1 | 0 |
| Positive | 202 | 66 | 32 | 12 | 3 | 1 | 0 |

**Supplementary Figure S5. Kaplan–Meier estimates of PFS according to PR status.**

PFS, progression-free survival; PR, progesterone receptor.

| PR Status | Median PFS (months) |  | Log-rank *P* value | Log-rank *P* value (overall) |
| --- | --- | --- | --- | --- |
| Negative | 7 | Negative vs High | 0.157 | 0.084 |
| Low | 7 | Low vs High | 0.108 |  |
| High | 7 | - |  |  |


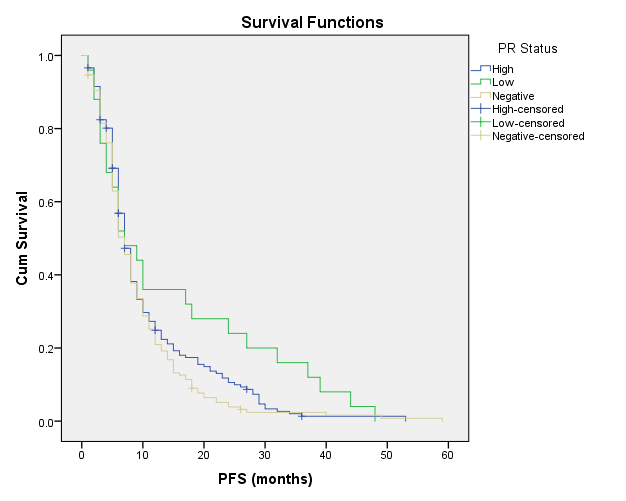


| No. at risk | | | | | | | |
| --- | --- | --- | --- | --- | --- | --- | --- |
| Months | 0 | 10 | 20 | 30 | 40 | 50 | 60 |
| Negative | 168 | 56 | 12 | 3 | 3 | 1 | 0 |
| Low | 25 | 11 | 7 | 5 | 2 | 0 | 0 |
| High | 177 | 55 | 25 | 7 | 1 | 1 | 0 |

**Supplementary Figure S6. Kaplan–Meier estimates of PFS according to PR status (three categories).**

PFS, progression-free survival; PR, progesterone receptor.

| HR Status | Median PFS (months) |  | Log-rank *P* value | Log-rank *P* value (overall) |
| --- | --- | --- | --- | --- |
| Negative | 6 | Negative vs High | 0.089 | 0.149 |
| Low | 7 | Low vs High | 0.455 |  |
| High | 7 | - |  |  |


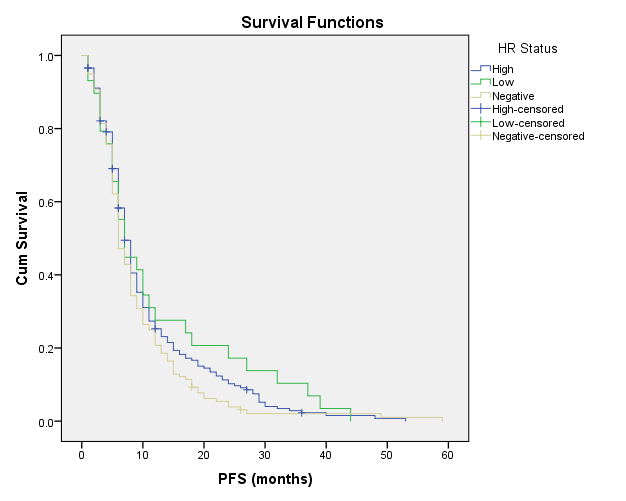


| No. at risk | | | | | | | |
| --- | --- | --- | --- | --- | --- | --- | --- |
| Months | 0 | 10 | 20 | 30 | 40 | 50 | 60 |
| Negative | 140 | 43 | 10 | 2 | 2 | 1 | 0 |
| Low | 29 | 12 | 6 | 4 | 1 | 0 | 0 |
| High | 203 | 67 | 28 | 9 | 3 | 1 | 0 |

**Supplementary Figure S7. Kaplan–Meier estimates of PFS according to HR status (three categories).**

HR, hormonal receptor; PFS, progression-free survival.

| HR Status | Median PFS (months) | Log-rank *P* value |
| --- | --- | --- |
| Negative | 6 | 0.068 |
| Positive | 7 |  |


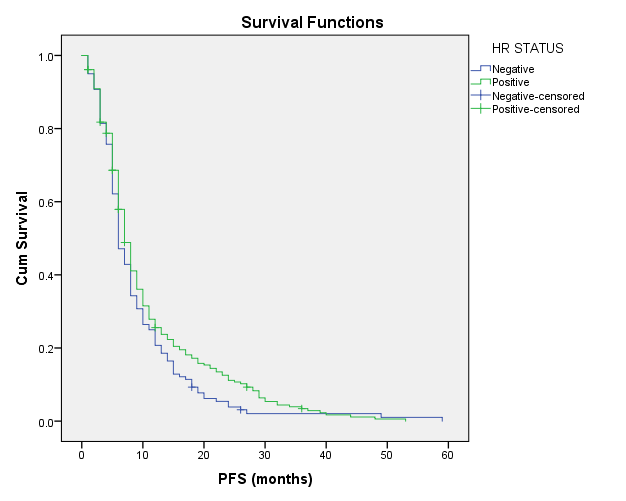


| No. at risk | | | | | | | |
| --- | --- | --- | --- | --- | --- | --- | --- |
| Months | 0 | 10 | 20 | 30 | 40 | 50 | 60 |
| Negative | 140 | 43 | 10 | 2 | 2 | 1 | 0 |
| Positive | 232 | 79 | 34 | 13 | 4 | 1 | 0 |

**Supplementary Figure S8. Kaplan–Meier estimates of PFS according to HR status.**

HR, hormonal receptor; PFS, progression-free survival.

| HER2 testing | Median PFS (months) | Comparison | Log-rank *P* value | Log-rank *P* value (overall) |
| --- | --- | --- | --- | --- |
| 0 | 7 | 0 Vs +3 | 0.304 | 0.802 |
| +1 | 7 | +1 vs +3 | 0.652 |  |
|  |  | +1 vs +2 | 0.780 |  |
| +2 | 7 | +2 vs +3 | 0.584 |  |
| +3 | 7 | - |  |  |


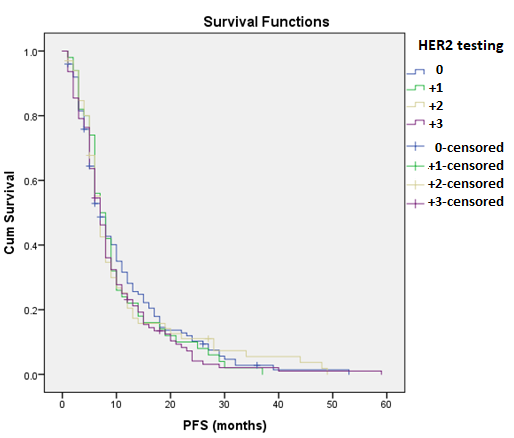


| No. at risk | | | | | | | |
| --- | --- | --- | --- | --- | --- | --- | --- |
| Months | 0 | 10 | 20 | 30 | 40 | 50 | 60 |
| 0 | 125 | 47 | 16 | 6 | 1 | 1 | 0 |
| +1 | 50 | 16 | 6 | 2 | 0 | 0 | 0 |
| +2 | 66 | 19 | 9 | 4 | 3 | 0 | 0 |
| +3 | 110 | 35 | 12 | 2 | 2 | 1 | 0 |

**Supplementary Figure S9. Kaplan–Meier estimates of PFS according to HER2 status.**

HER2, human epidermal growth factor receptor 2; PFS, progression-free survival.

| HER2 Status | Median PFS (months) | Log-rank *P* value |
| --- | --- | --- |
| Negative | 7 | 0.368 |
| Positive | 7 |  |


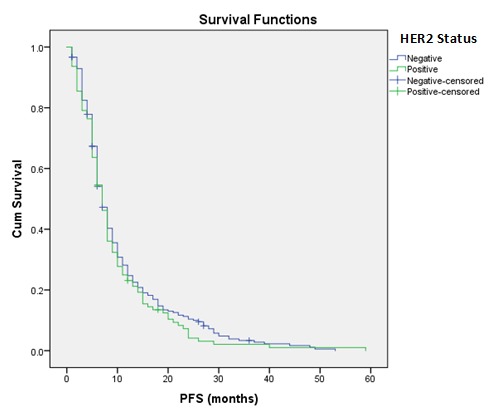


| No. at risk | | | | | | | |
| --- | --- | --- | --- | --- | --- | --- | --- |
| Months | 0 | 10 | 20 | 30 | 40 | 50 | 60 |
| Negative | 241 | 82 | 31 | 12 | 4 | 1 | 0 |
| Positive | 110 | 35 | 12 | 2 | 2 | 1 | 0 |

**Supplementary Figure S10. Kaplan–Meier estimates of PFS according to HER2 status (two categories).**

HER2, human epidermal growth factor receptor 2; PFS, progression-free survival.

| HR/HER2 status | Median PFS (months) |  | Log-rank *P* value | Log-rank *P* value (overall) |
| --- | --- | --- | --- | --- |
| HR-positive/HER2-negative | 8 | HR-positive /HER2-negative vs TNBC | **0.012** | 0.056 |
| HR-positive/HER2-positive | 7 | HR-positive /HER2-positive vs TNBC | 0.767 |  |
| HR-negative/HER2-positive | 8 | HR-negative /HER2-positive vs TNBC | 0.312 |  |
| TNBC | 6 | - |  |  |


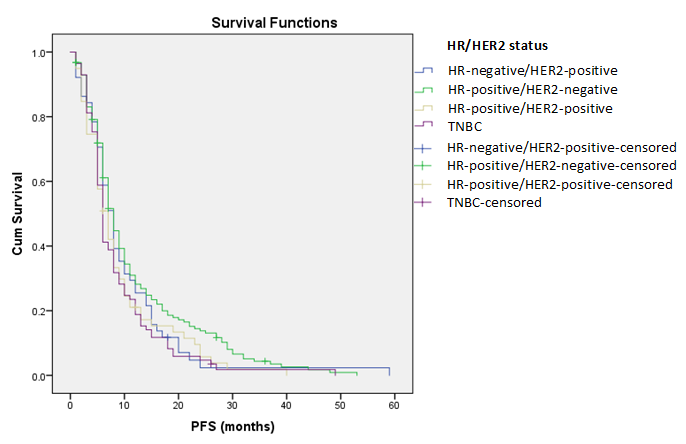


| No. at risk | | | | | | | |
| --- | --- | --- | --- | --- | --- | --- | --- |
| Months | 0 | 10 | 20 | 30 | 40 | 50 | 60 |
| HR-positive /HER2-negative | 155 | 57 | 26 | 11 | 3 | 1 | 0 |
| HR-positive /HER2-positive | 59 | 17 | 7 | 1 | 1 | 0 | 0 |
| HR-negative /HER2-positive | 51 | 18 | 5 | 1 | 1 | 1 | 0 |
| TNBC | 85 | 24 | 5 | 1 | 1 | 0 | 0 |

**Supplementary Figure S11. Kaplan–Meier estimates of PFS according to HR/HER2 status.**

HER2, human epidermal growth factor receptor 2; HR, hormonal receptor; PFS, progression-free survival; TNBC, triple-negative breast cancer.

| Grade status | Median PFS (months) | Log-rank *P* value |
| --- | --- | --- |
| G1 | 8 | 0.857 |
| G2 | 7 |  |
| G3 | 7 |  |


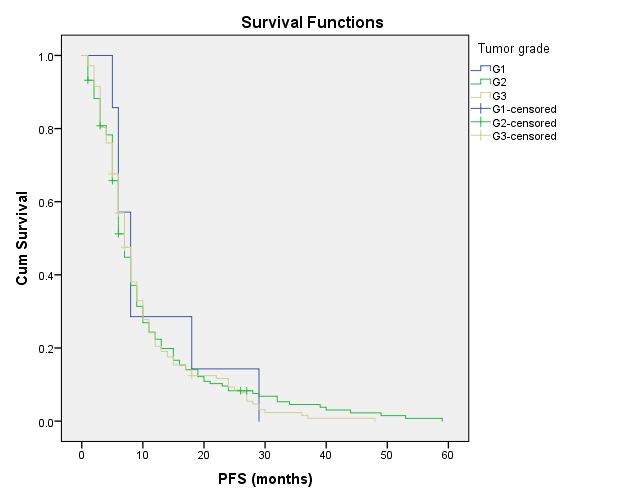


| No. at risk | | | | | | | |
| --- | --- | --- | --- | --- | --- | --- | --- |
| Months | 0 | 10 | 20 | 30 | 40 | 50 | 60 |
| G1 | 7 | 2 | 1 | 0 | 0 | 0 | 0 |
| G2 | 162 | 49 | 19 | 9 | 5 | 2 | 0 |
| G3 | 142 | 45 | 16 | 4 | 1 | 0 | 0 |

**Supplementary Figure S12. Kaplan–Meier estimates of PFS according to grade status.**

PFS, progression-free survival.

| Stage | Median PFS (months) | Log-rank *P* value |
| --- | --- | --- |
| 3 | 6 | 0.452 |
| 4 | 7 |  |


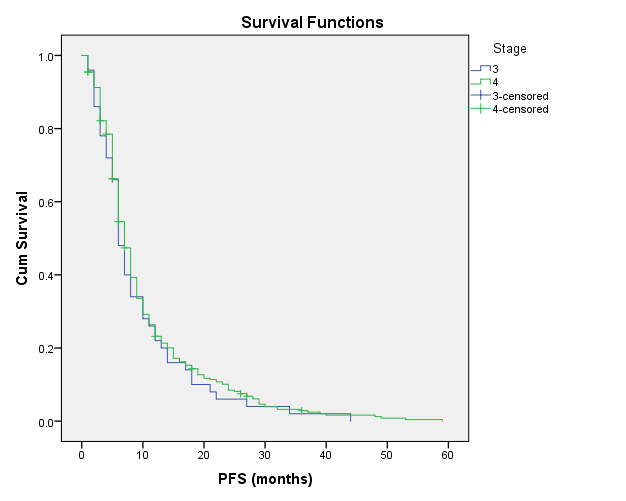


| No. at risk | | | | | | | |
| --- | --- | --- | --- | --- | --- | --- | --- |
| Months | 0 | 10 | 20 | 30 | 40 | 50 | 60 |
| 3 | 50 | 17 | 5 | 2 | 1 | 0 | 0 |
| 4 | 332 | 107 | 39 | 13 | 5 | 2 | 0 |

**Supplementary Figure S13. Kaplan–Meier estimates of PFS according to stage.**

PFS, progression-free survival.

| Metastatic site | Median PFS (months) | Log-rank *P* value (overall) |
| --- | --- | --- |
| Bone | 8 | **0.003** |
| Brain | 5 |  |
| Liver | 7 |  |
| Local | 6 |  |
| Lung | 5 |  |
| Multiple | 8 |  |
| Other M. | 7 |  |


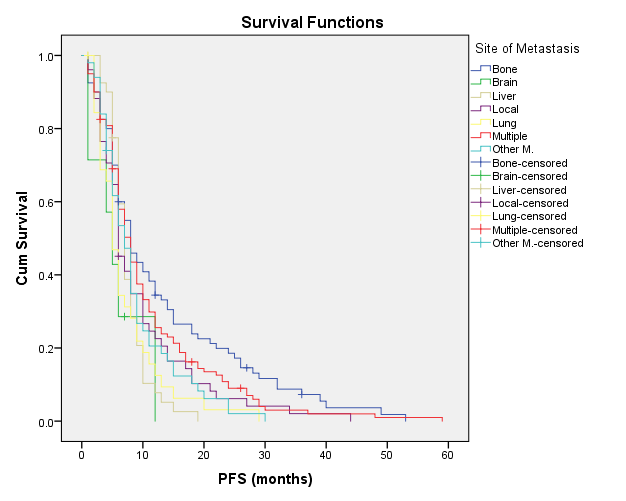


| No. at risk | | | | | | | |
| --- | --- | --- | --- | --- | --- | --- | --- |
| Months | 0 | 10 | 20 | 30 | 40 | 50 | 60 |
| Bone | 80 | 34 | 17 | 8 | 3 | 1 | 0 |
| Brain | 7 | 1 | 0 | 0 | 0 | 0 | 0 |
| Liver | 41 | 8 | 0 | 0 | 0 | 0 | 0 |
| Local | 51 | 17 | 5 | 2 | 1 | 0 | 0 |
| Lung | 33 | 7 | 2 | 0 | 0 | 0 | 0 |
| Multiple | 120 | 44 | 16 | 4 | 2 | 1 | 0 |
| Other M | 50 | 13 | 4 | 1 | 0 | 0 | 0 |

**Supplementary Figure S14. Kaplan–Meier estimates of PFS according to metastatic site.**

PFS, progression-free survival; M, metastasis.

| No. of metastasis site | Median PFS (months) | Log-rank *P* value |
| --- | --- | --- |
| Multiple | 8 | 0.284 |
| Single | 7 |  |


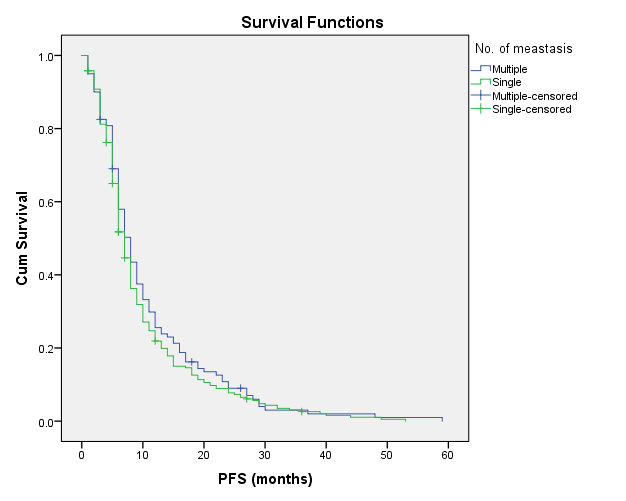


| No. at risk | | | | | | | |
| --- | --- | --- | --- | --- | --- | --- | --- |
| Months | 0 | 10 | 20 | 30 | 40 | 50 | 60 |
| Multiple | 120 | 44 | 16 | 4 | 2 | 1 | 0 |
| Single | 262 | 80 | 28 | 11 | 4 | 1 | 0 |

**Supplementary Figure S15. Kaplan–Meier estimates of PFS according to no. of metastasis site (multiple vs single).**

PFS, progression-free survival.

| No. of metastasis site | Median PFS (months) | Log-rank *P* value |
| --- | --- | --- |
| >2 | 9 | 0.187 |
| ≤2 | 7 |  |


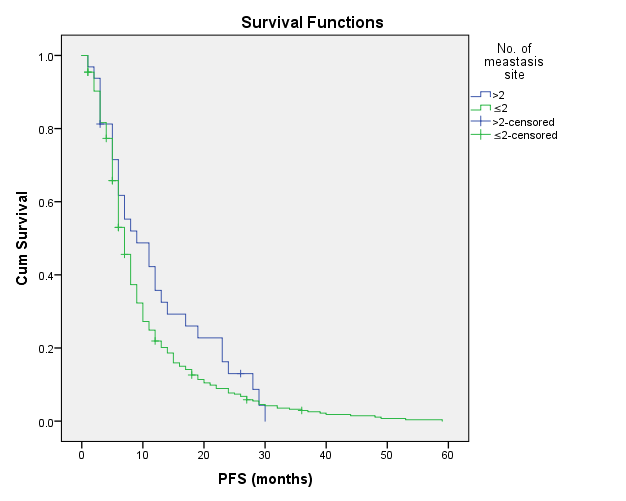


| No. at risk | | | | | | | |
| --- | --- | --- | --- | --- | --- | --- | --- |
| Months | 0 | 10 | 20 | 30 | 40 | 50 | 60 |
| >2 | 32 | 15 | 7 | 1 | 0 | 0 | 0 |
| ≤2 | 350 | 109 | 37 | 14 | 6 | 2 | 0 |

**Supplementary Figure S16. Kaplan–Meier estimates of PFS according to no. of metastasis site (>2 vs ≤2).**

PFS, progression-free survival.

| Site of metastases | Median PFS (months) | Log-rank *P* value |
| --- | --- | --- |
| Liver | 7 | 0.130 |
| Other | 7 |  |


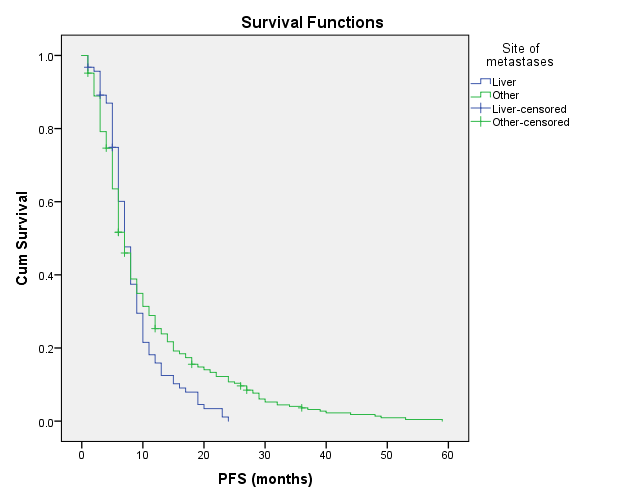


| No. at risk | | | | | | | |
| --- | --- | --- | --- | --- | --- | --- | --- |
| Months | 0 | 10 | 20 | 30 | 40 | 50 | 60 |
| Liver | 93 | 26 | 4 | 0 | 0 | 0 | 0 |
| Other | 289 | 98 | 40 | 15 | 6 | 2 | 0 |

**Supplementary Figure S17. Kaplan–Meier estimates of PFS according to liver metastasis.**

PFS, progression-free survival.

| Site of metastases | Median PFS (months) | Log-rank *P* value |
| --- | --- | --- |
| Lung | 6 | 0.986 |
| Other | 7 |  |


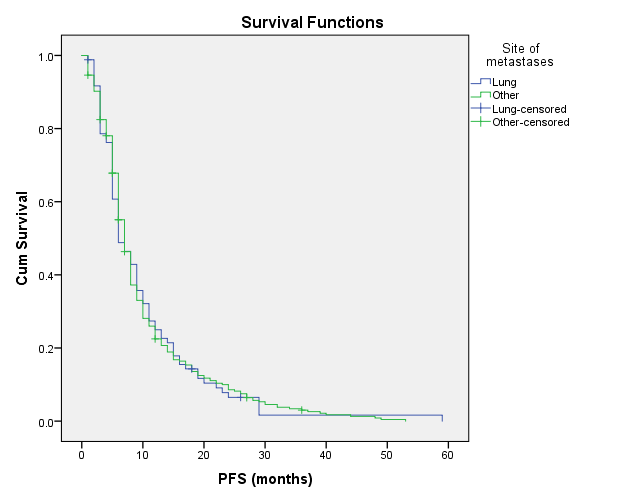


| No. at risk | | | | | | | |
| --- | --- | --- | --- | --- | --- | --- | --- |
| Months | 0 | 10 | 20 | 30 | 40 | 50 | 60 |
| Lung | 85 | 30 | 9 | 1 | 1 | 1 | 0 |
| Other | 297 | 94 | 35 | 14 | 5 | 1 | 0 |

**Supplementary Figure S18. Kaplan–Meier estimates of PFS according to lung metastasis.**

PFS, progression-free survival.

| Site of metastases | Median PFS (months) | Log-rank *P* value |
| --- | --- | --- |
| Local | 6 | 0.407 |
| Other | 7 |  |


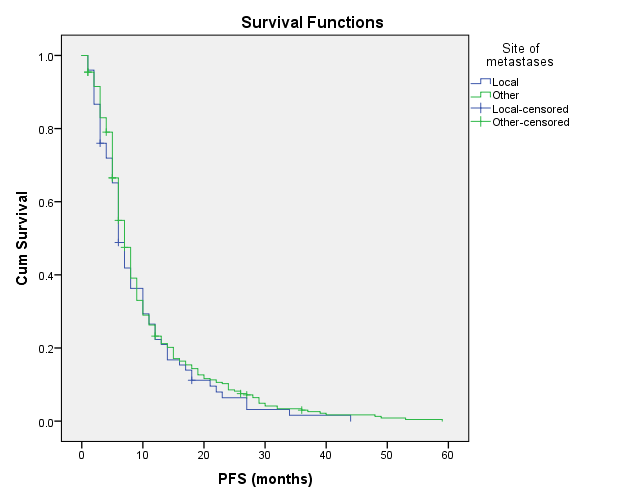


| No. at risk | | | | | | | |
| --- | --- | --- | --- | --- | --- | --- | --- |
| Months | 0 | 10 | 20 | 30 | 40 | 50 | 60 |
| Local | 75 | 26 | 7 | 2 | 1 | 0 | 0 |
| Other | 307 | 98 | 37 | 13 | 5 | 2 | 2 |

**Supplementary Figure S19. Kaplan–Meier estimates of PFS according to local unresectable disease.**

PFS, progression-free survival.

| Site of metastases | Median PFS (months) | Log-rank *P* value |
| --- | --- | --- |
| Node | 7 | 0.830 |
| Other | 7 |  |


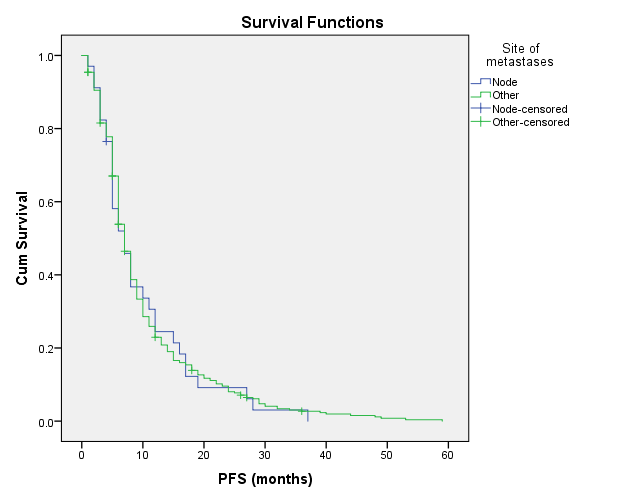


| No. at risk | | | | | | | |
| --- | --- | --- | --- | --- | --- | --- | --- |
| Months | 0 | 10 | 20 | 30 | 40 | 50 | 60 |
| Node | 34 | 12 | 3 | 1 | 0 | 0 | 0 |
| Other | 348 | 112 | 41 | 14 | 6 | 2 | 0 |

**Supplementary Figure S20. Kaplan–Meier estimates of PFS according to node metastasis.**

PFS, progression-free survival.

| Site of metastases | Median PFS (months) | Log-rank *P* value |
| --- | --- | --- |
| Other | 7 | 0.483 |
| Pleural | 8 |  |


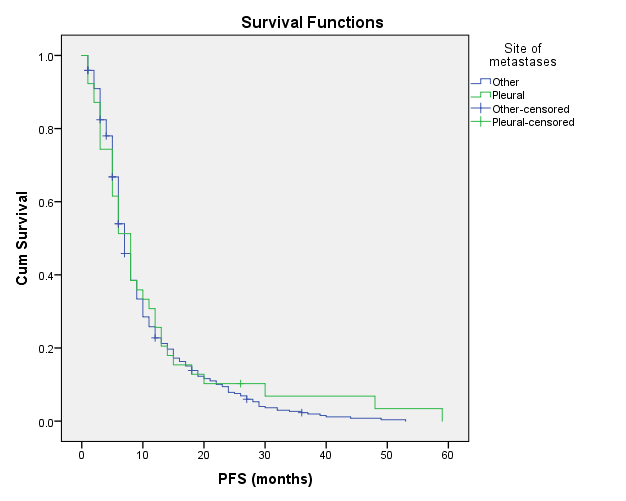


| No. at risk | | | | | | | |
| --- | --- | --- | --- | --- | --- | --- | --- |
| Months | 0 | 10 | 20 | 30 | 40 | 50 | 60 |
| Other | 39 | 14 | 5 | 3 | 2 | 1 | 0 |
| Pleural | 343 | 110 | 39 | 12 | 4 | 1 | 0 |

**Supplementary Figure S21. Kaplan–Meier estimates of PFS according to pleural metastasis.**

PFS, progression-free survival.

| Site of metastases | Median PFS (months) | Log-rank *P* value |
| --- | --- | --- |
| Other | 7 | 0.201 |
| Skin | 7 |  |


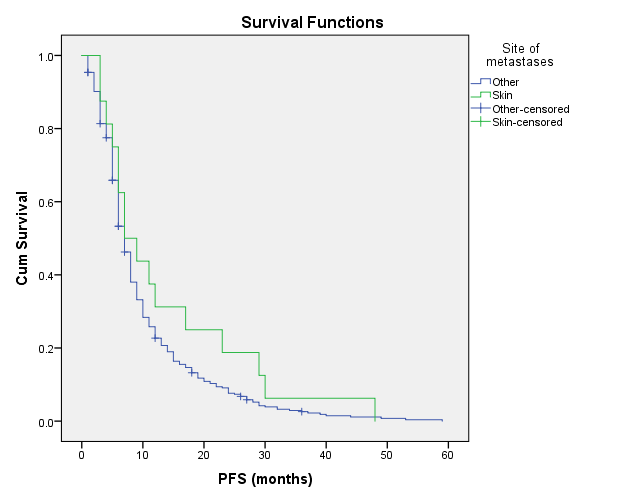


| No. at risk | | | | | | | |
| --- | --- | --- | --- | --- | --- | --- | --- |
| Months | 0 | 10 | 20 | 30 | 40 | 50 | 60 |
| Other | 366 | 117 | 40 | 13 | 5 | 2 | 0 |
| Skin | 16 | 7 | 4 | 2 | 1 | 0 | 0 |

**Supplementary Figure S22. Kaplan–Meier estimates of PFS according to skin metastasis.**

PFS, progression-free survival.

| Site of metastases | Median PFS (months) | Log-rank *P* value |
| --- | --- | --- |
| Other | 7 | 0.263 |
| Other B. | 9 |  |


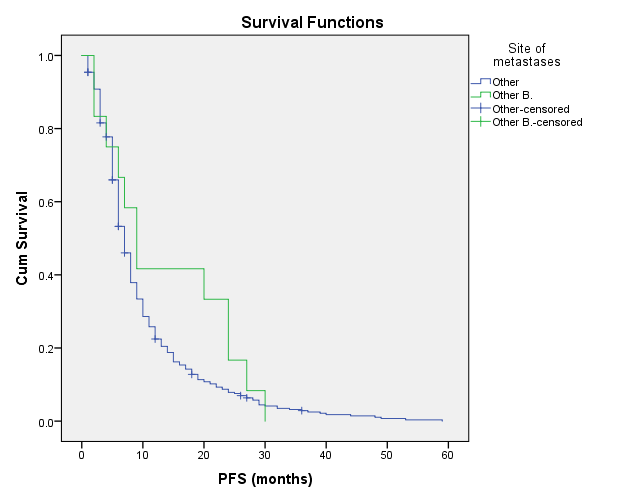


| No. at risk | | | | | | | |
| --- | --- | --- | --- | --- | --- | --- | --- |
| Months | 0 | 10 | 20 | 30 | 40 | 50 | 60 |
| Other | 370 | 119 | 39 | 14 | 6 | 2 | 0 |
| Other B. | 12 | 5 | 5 | 1 | 0 | 0 | 0 |

**Supplementary Figure S23. Kaplan–Meier estimates of PFS according to the other breast metastasis.**

PFS, progression-free survival; B, breast.

| Site of metastases | Median PFS (months) | Log-rank *P* value |
| --- | --- | --- |
| Ascites | 6 | 0.970 |
| Other | 7 |  |


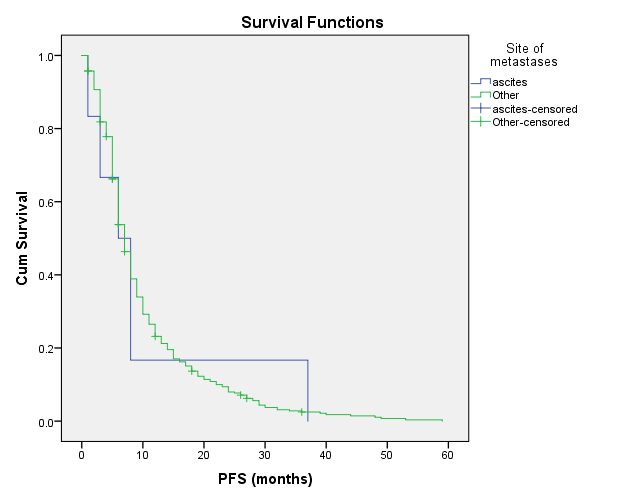


| No. at risk | | | | | | | |
| --- | --- | --- | --- | --- | --- | --- | --- |
| Months | 0 | 10 | 20 | 30 | 40 | 50 | 60 |
| Ascites | 6 | 1 | 1 | 1 | 0 | 0 | 0 |
| Other | 376 | 123 | 43 | 14 | 6 | 2 | 1 |

**Supplementary Figure S24. Kaplan–Meier estimates of PFS according to ascites.**

PFS, progression-free survival.

| Site of metastases | Median PFS (months) | Log-rank *P* value |
| --- | --- | --- |
| Other | 7 | 0.113 |
| Pericar. | 4 |  |


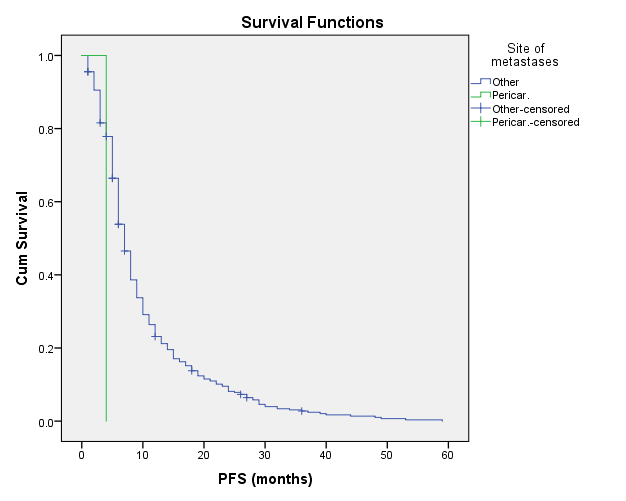


| No. at risk | | | | | | | |
| --- | --- | --- | --- | --- | --- | --- | --- |
| Months | 0 | 10 | 20 | 30 | 40 | 50 | 60 |
| Other | 381 | 124 | 44 | 15 | 6 | 2 | 0 |
| Pericar. | 1 | 0 | 0 | 0 | 0 | 0 | 0 |

**Supplementary Figure S25. Kaplan–Meier estimates of PFS according to pericardial metastasis.**

PFS, progression-free survival; Pericar, pericardial.

| Site of metastases | Median PFS (months) | Log-rank *P* value |
| --- | --- | --- |
| Other | 7 | 0.305 |
| Spleen | 5 |  |


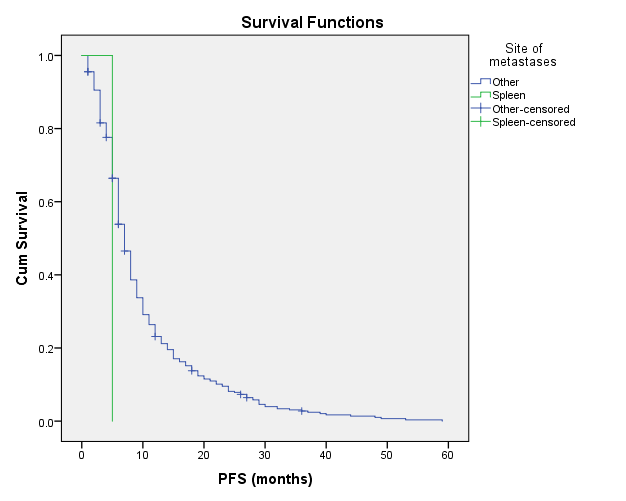


| No. at risk | | | | | | | |
| --- | --- | --- | --- | --- | --- | --- | --- |
| Months | 0 | 10 | 20 | 30 | 40 | 50 | 60 |
| Other | 381 | 124 | 44 | 15 | 6 | 2 | 0 |
| Spleen | 1 | 0 | 0 | 0 | 0 | 0 | 0 |

**Supplementary Figure S26. Kaplan–Meier estimates of PFS according to spleen metastasis.**

PFS, progression-free survival.

| CEA levels at diagnosis | Median PFS (months) | Log-rank *P* value |
| --- | --- | --- |
| Normal | 6 | 0.937 |
| High | 6 |  |


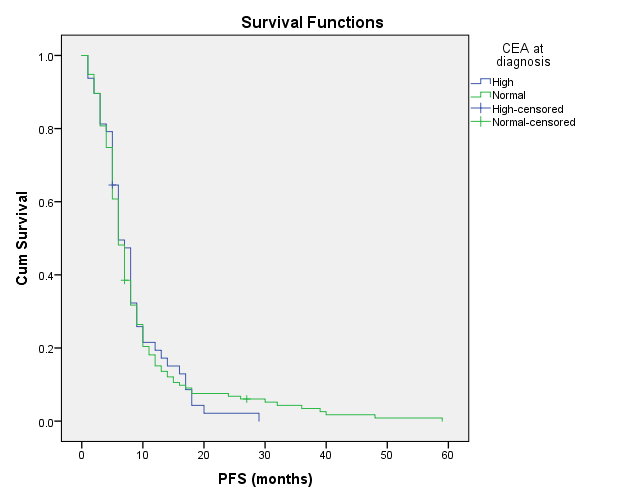


| No. at risk | | | | | | | |
| --- | --- | --- | --- | --- | --- | --- | --- |
| Months | 0 | 10 | 20 | 30 | 40 | 50 | 60 |
| Normal | 135 | 35 | 10 | 7 | 3 | 1 | 0 |
| High | 48 | 12 | 2 | 0 | 0 | 0 | 0 |

**Supplementary Figure S27. Kaplan–Meier estimates of PFS according to CEA levels** **at diagnosis.**

CEA, carcinoembryonic antigen; PFS, progression-free survival.

| CA15-3 levels at diagnosis | Median PFS (months) | Log-rank *P* value |
| --- | --- | --- |
| Normal | 6 | 0.853 |
| High | 6 |  |


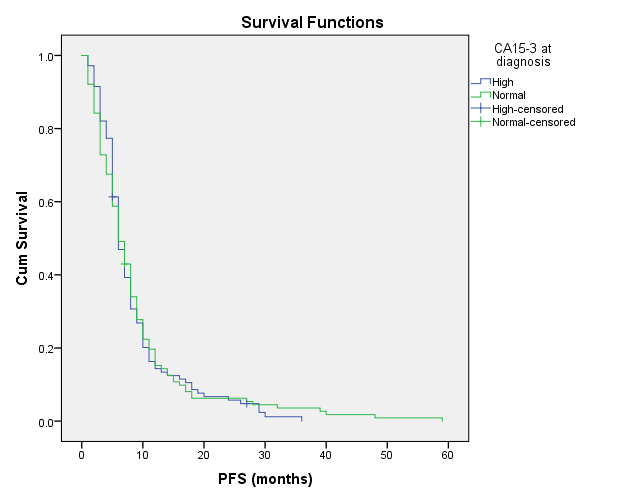


| No. at risk | | | | | | | |
| --- | --- | --- | --- | --- | --- | --- | --- |
| Months | 0 | 10 | 20 | 30 | 40 | 50 | 60 |
| Normal | 114 | 31 | 7 | 5 | 3 | 1 | 0 |
| High | 106 | 28 | 8 | 2 | 0 | 0 | 0 |

**Supplementary Figure S28. Kaplan–Meier estimates of PFS according to CA15-3 levels** **at diagnosis.**

CA15-3, cancer antigen 15-3; PFS, progression-free survival.

| First-line treatment | Median PFS (months) |  | Log-rank *P* value | Log-rank *P* value (overall) |
| --- | --- | --- | --- | --- |
| Anti-HER2 | 6 | Anti-HER2 vs ET | **0.002** | **<0.0001** |
| Chemotherapy | 7 | Chemotherapy vs ET | **<0.0001** |  |
| ET | 19 | - | - |  |


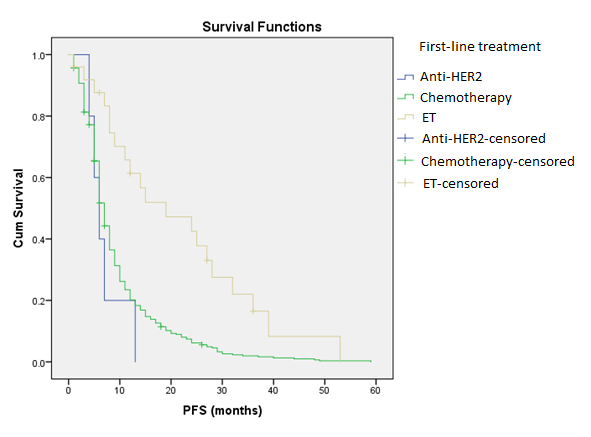


| No. at risk | | | | | | | |
| --- | --- | --- | --- | --- | --- | --- | --- |
| Months | 0 | 10 | 20 | 30 | 40 | 50 | 60 |
| Anti-HER2 | 5 | 1 | 0 | 0 | 0 | 0 | 0 |
| Chemotherapy | 343 | 104 | 33 | 10 | 5 | 1 | 0 |
| ET | 25 | 16 | 10 | 5 | 1 | 1 | 0 |

**Supplementary Figure S29. Kaplan–Meier estimates of PFS according to first-line treatment (ET vs anti-HER2 vs other).**

HER2, human epidermal growth factor receptor 2; ET, endocrine therapy; PFS, progression-free survival.

| First-line treatment | Median PFS (months) | Log-rank *P* value (overall) |
| --- | --- | --- |
| Anthracycline-based | 7 | **<0.0001** |
| Anti-HER2 | 6 |  |
| AT | 6 |  |
| ET | 19 |  |
| Other | 7 |  |
| TXN-based | 7 |  |


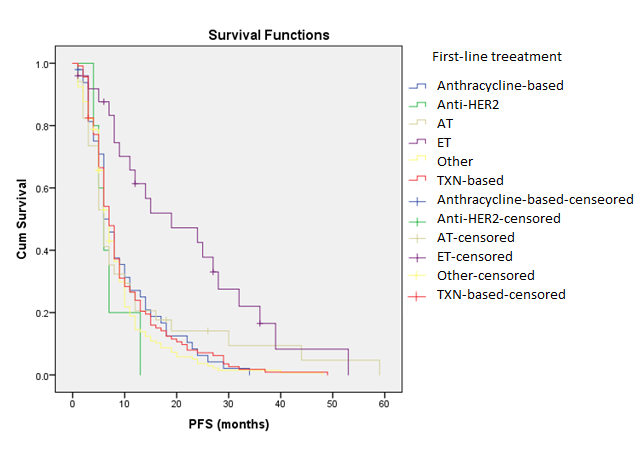


| No. at risk | | | | | | | |
| --- | --- | --- | --- | --- | --- | --- | --- |
| Months | 0 | 10 | 20 | 30 | 40 | 50 | 60 |
| Anthracycline-based | 49 | 17 | 6 | 1 | 0 | 0 | 0 |
| Anti-HER2 | 5 | 1 | 0 | 0 | 0 | 0 | 0 |
| AT | 34 | 11 | 4 | 3 | 2 | 1 | 0 |
| ET | 25 | 16 | 10 | 5 | 1 | 1 | 0 |
| Others* | 146 | 41 | 10 | 2 | 2 | 1 | 0 |
| TXN-based | 114 | 35 | 13 | 4 | 1 | 0 | 0 |

**Supplementary Figure S30. Kaplan–Meier estimates of PFS according to first-line treatment.**

*Others include: capecitabine (single agent), cyclophosphamide + methotrexate + fluorouracil, gemcitabine + vinorelbine, platinum+ gemcitabine, platinum + vinorelbine, vinorelbine (single agent), vinorelbine + capecitabine.

AT, doxorubicin and docetaxel; HER2, human epidermal growth factor receptor 2; ET, endocrine therapy; PFS, progression-free survival; TXN, taxane.

1. **Overall survival (OS) analysis according to clinicopathological features for patients with advanced breast cancer (423 patients)**

| Mean ± SD OS (months) | Median OS (months) | Range | 95% CI |
| --- | --- | --- | --- |
| 22.16 | 16 | 1-102 | 14.357-17.643 |


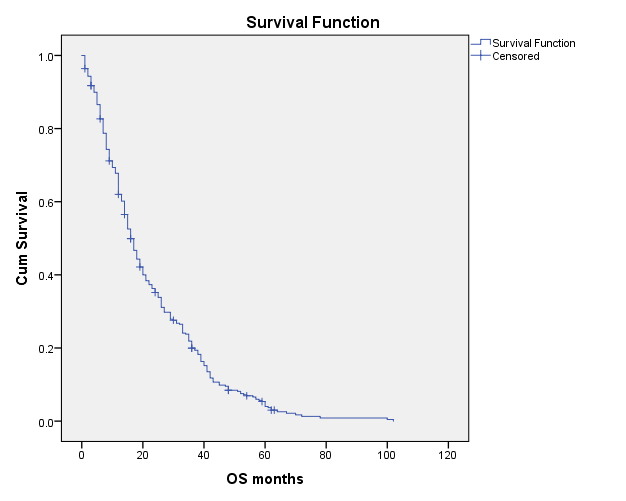


| Months | 0 | 20 | 40 | 60 | 80 | 100 | 120 |
| --- | --- | --- | --- | --- | --- | --- | --- |
| No. at risk | 388 | 157 | 58 | 16 | 2 | 2 | 0 |

**Supplementary Figure S31. Kaplan–Meier estimate of OS of patients with advanced breast cancer.**

CI, confidence interval; OS, overall survival; SD, standard deviation.

| Menopause status | Median OS (months) | Log-rank *P* value |
| --- | --- | --- |
| Post | 16 | 0.523 |
| Pre | 17 |  |


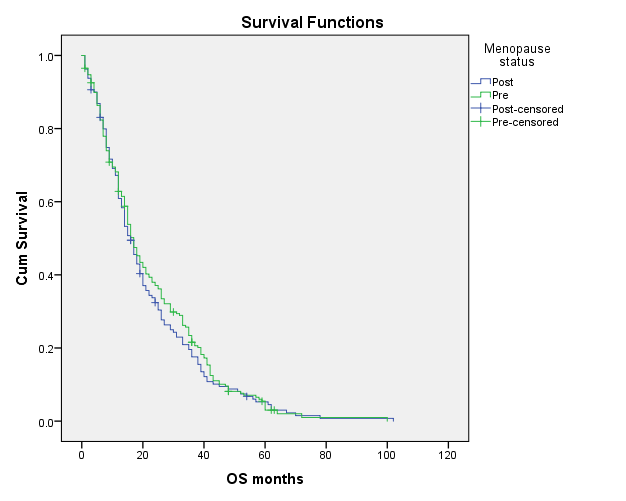


| No. at risk | | | | | | | |
| --- | --- | --- | --- | --- | --- | --- | --- |
| Months | 0 | 20 | 40 | 60 | 80 | 100 | 120 |
| Post | 160 | 61 | 20 | 7 | 1 | 1 | 0 |
| Pre | 228 | 96 | 38 | 9 | 1 | 1 | 0 |

**Supplementary Figure S32. Kaplan–Meier estimates of OS according to menopause status.**

OS, overall survival.

| ER Status | Median OS (months) | Log-rank *P* value |
| --- | --- | --- |
| Negative | 16 | 0.158 |
| Positive | 17 |  |


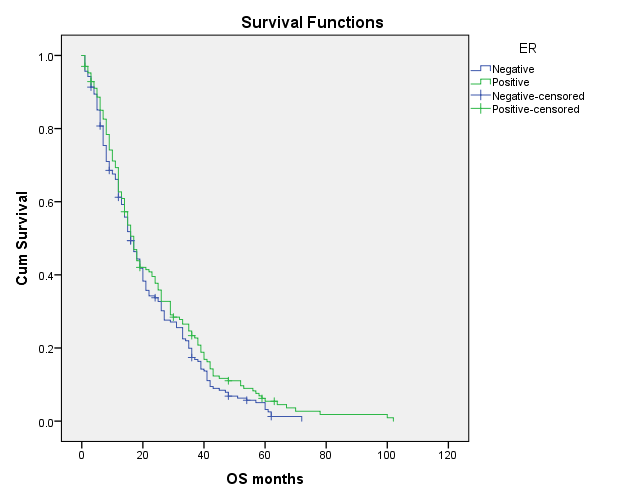


| No. at risk | | | | | | | |
| --- | --- | --- | --- | --- | --- | --- | --- |
| Months | 0 | 20 | 40 | 60 | 80 | 100 | 120 |
| Negative | 208 | 83 | 27 | 8 | 0 | 0 | 0 |
| Positive | 168 | 68 | 29 | 8 | 2 | 2 | 0 |

**Supplementary Figure S33. Kaplan–Meier estimates of OS according to ER status.**

ER, estrogen receptor; OS, overall survival.

| ER Status | Median OS (months) |  | Log-rank *P* value | Log-rank *P* value (overall) |
| --- | --- | --- | --- | --- |
| Negative | 16 | Negative vs High | 0.153 | 0.353 |
| Low | 16 | Low vs High | 0.835 |  |
| High | 17 | - |  |  |


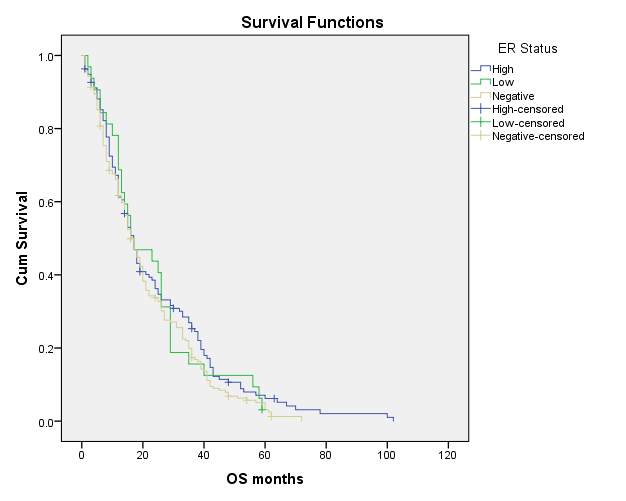


| No. at risk | | | | | | | |
| --- | --- | --- | --- | --- | --- | --- | --- |
| Months | 0 | 20 | 40 | 60 | 80 | 100 | 120 |
| Negative | 208 | 83 | 27 | 8 | 0 | 0 | 0 |
| Low | 32 | 15 | 5 | 0 | 0 | 0 | 0 |
| High | 136 | 53 | 24 | 8 | 2 | 2 | 0 |

**Supplementary Figure S34. Kaplan–Meier estimates of PFS according to ER status (three categories).**

ER, estrogen receptor; OS, overall survival.

| PR Status | Median OS (months) | Log-rank *P* value |
| --- | --- | --- |
| Negative | 15 | **0.020** |
| Positive | 17 |  |


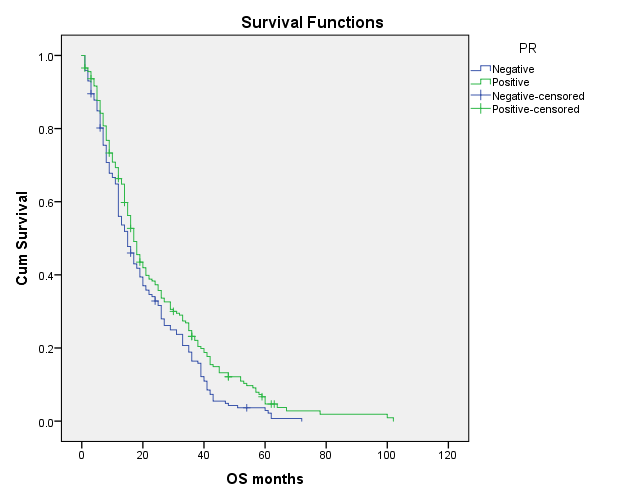


| No. at risk | | | | | | | |
| --- | --- | --- | --- | --- | --- | --- | --- |
| Months | 0 | 20 | 40 | 60 | 80 | 100 | 120 |
| Negative | 172 | 66 | 20 | 5 | 0 | 0 | 0 |
| Positive | 204 | 84 | 36 | 10 | 2 | 2 | 0 |

**Supplementary Figure S35. Kaplan–Meier estimates of OS according to PR status.**

OS, overall survival; PR, progesterone receptor.

| PR Status | Median OS (months) |  | Log-rank *P* value | Log-rank *P* value (overall) |
| --- | --- | --- | --- | --- |
| Negative | 15 | Negative vs High | 0.073 | **0.025** |
|  |  | Negative vs Low | **0.03** |  |
| Low | 23 | Low vs High | 0.137 |  |
| High | 17 | - |  |  |


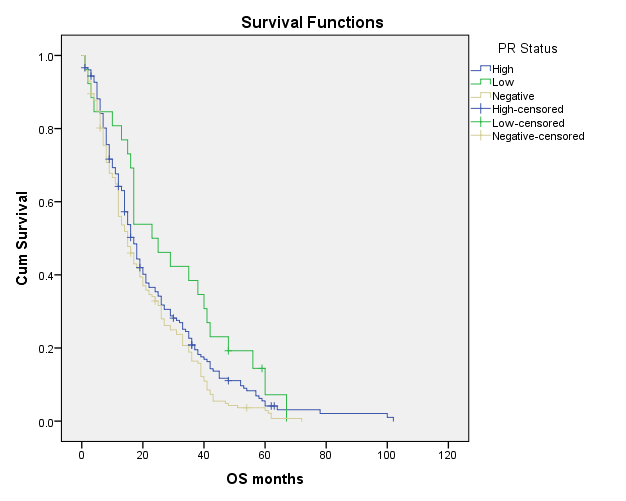


| No. at risk | | | | | | | |
| --- | --- | --- | --- | --- | --- | --- | --- |
| Months | 0 | 20 | 40 | 60 | 80 | 100 | 120 |
| Negative | 172 | 66 | 20 | 5 | 0 | 0 | 0 |
| Low | 26 | 14 | 9 | 2 | 0 | 0 | 0 |
| High | 178 | 70 | 27 | 8 | 2 | 2 | 0 |

**Supplementary Figure S36. Kaplan–Meier estimates of OS according to PR status (three categories).**

OS, overall survival; PR, progesterone receptor.

| HR Status | Median OS (months) |  | Log-rank *P* value | Log-rank *P* value (overall) |
| --- | --- | --- | --- | --- |
| Negative | 15 | Negative vs High | 0.056 | 0.091 |
|  |  | Negative vs Low | 0.121 |  |
| Low | 23 | Low vs High | 0.565 |  |
| High | 17 | - |  |  |


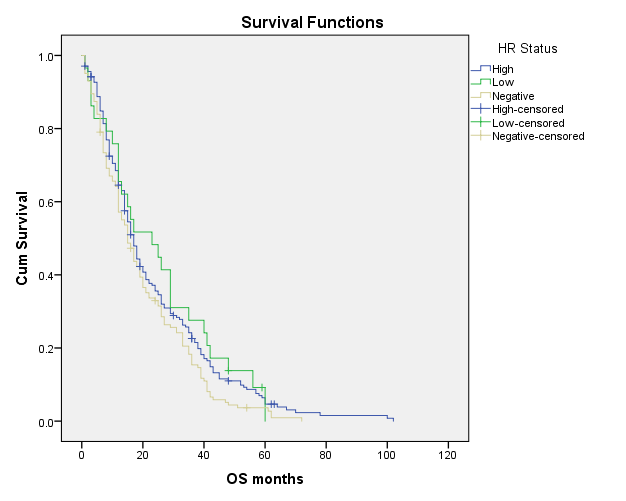


| No. at risk | | | | | | | |
| --- | --- | --- | --- | --- | --- | --- | --- |
| Months | 0 | 20 | 40 | 60 | 80 | 100 | 120 |
| Negative | 143 | 55 | 16 | 4 | 0 | 0 | 0 |
| Low | 29 | 15 | 8 | 1 | 0 | 0 | 0 |
| High | 206 | 82 | 33 | 11 | 2 | 2 | 0 |

**Supplementary Figure S37. Kaplan–Meier estimates of OS according to HR status (three categories).**

HR, hormonal receptor; OS, overall survival.

| HER2 testing | Median OS (months) | Comparison | Log-rank *P* value | Log-rank *P* value (overall) |
| --- | --- | --- | --- | --- |
| 0 | 16 | 0 Vs +3 | 0.635 | 0.749 |
| +1 | 17 | +1 vs +3 | 0.396 |  |
|  |  | +1 vs +2 | 0.888 |  |
| +2 | 17 | +2 vs +3 | 0.354 |  |
| +3 | 16 | - | - |  |


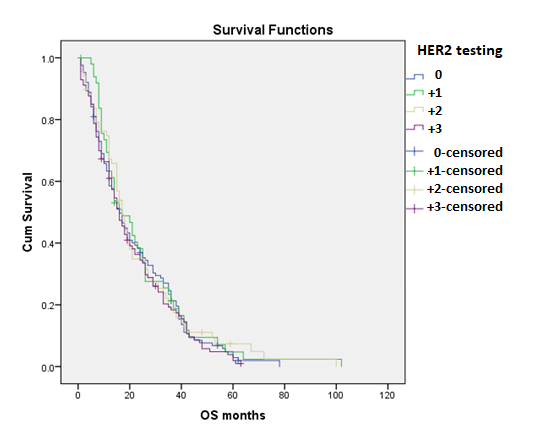


| No. at risk | | | | | | | |
| --- | --- | --- | --- | --- | --- | --- | --- |
| Months | 0 | 20 | 40 | 60 | 80 | 100 | 120 |
| 0 | 126 | 54 | 18 | 5 | 0 | 0 | 0 |
| +1 | 50 | 23 | 7 | 2 | 1 | 1 | 0 |
| +2 | 68 | 25 | 10 | 3 | 1 | 1 | 0 |
| +3 | 113 | 44 | 17 | 4 | 0 | 0 | 0 |

**Supplementary Figure S38. Kaplan–Meier estimates of OS according to HER2 status.**

HER2, human epidermal growth factor receptor 2; OS, overall survival.

| HER2 status | Median OS (months) | Log-rank *P* value |
| --- | --- | --- |
| Negative | 17 | 0.375 |
| Positive | 16 |  |


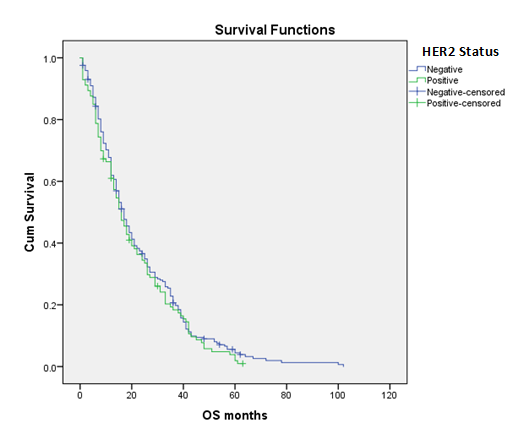


| No. at risk | | | | | | | |
| --- | --- | --- | --- | --- | --- | --- | --- |
| Months | 0 | 20 | 40 | 60 | 80 | 100 | 120 |
| Negative | 244 | 102 | 35 | 10 | 2 | 2 | 0 |
| Positive | 113 | 44 | 17 | 4 | 0 | 0 | 0 |

**Supplementary Figure S39. Kaplan–Meier estimates of OS according to HER2 status (two categories).**

HER2, human epidermal growth factor receptor 2; OS, overall survival.

| HR/HER2 status | Median OS (months) |  | Log-rank P value | Log-rank *P* value (overall) |
| --- | --- | --- | --- | --- |
| HR-positive /HER2-negative | 18 | HR-positive /HER2-negative vs TNBC | **0.040** | 0.151 |
| HR-positive /HER2-positive | 16 | HR-positive /HER2-positive vs TNBC | 0.497 |  |
| HR-negative /HER2-positive | 15 | HR-negative /HER2-positive vs TNBC | 0.821 |  |
| TNBC | 15 | - | - |  |


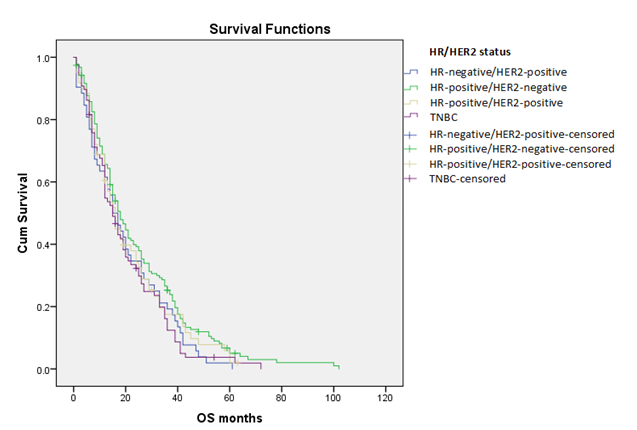


| No. at risk | | | | | | | |
| --- | --- | --- | --- | --- | --- | --- | --- |
| Months | 0 | 20 | 40 | 60 | 80 | 100 | 120 |
| HR-positive /HER2-negative | 156 | 70 | 28 | 8 | 2 | 2 | 0 |
| HR-positive /HER2-positive | 61 | 22 | 9 | 3 | 0 | 0 | 0 |
| HR-negative /HER2-positive | 52 | 22 | 8 | 1 | 0 | 0 | 0 |
| TNBC | 87 | 32 | 7 | 2 | 0 | 0 | 0 |

**Supplementary Figure S40. Kaplan–Meier estimates of OS according to HR/HER2 status.**

HER2, human epidermal growth factor receptor 2; HR, hormonal receptor; TNBC, triple-negative breast cancer; OS, overall survival.

| HR/HER2 status | Median OS (months) | Log-rank *P* value |
| --- | --- | --- |
| Other | 17 | 0.106 |
| TNBC | 15 |  |


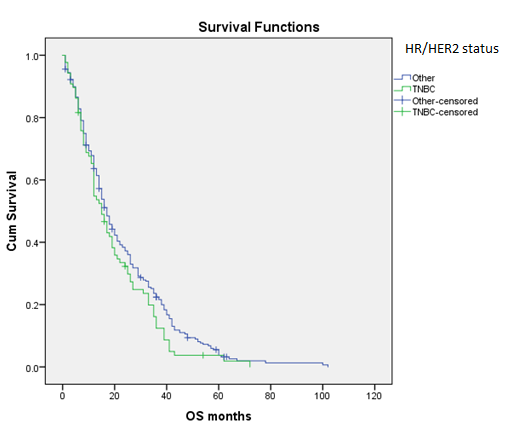


| No. at risk | | | | | | | |
| --- | --- | --- | --- | --- | --- | --- | --- |
| Months | 0 | 20 | 40 | 60 | 80 | 100 | 120 |
| Other | 269 | 114 | 45 | 12 | 2 | 2 | 0 |
| TNBC | 87 | 32 | 7 | 2 | 0 | 0 | 0 |

**Supplementary Figure S41. Kaplan–Meier estimates of OS according to HR/HER2 status (two categories).**

HER2, human epidermal growth factor receptor 2; HR, hormonal receptor; TNBC, triple-negative breast cancer; OS, overall survival.

| Grade | Median OS (months) |  | Log-rank *P* value | Log-rank *P* value (overall) |
| --- | --- | --- | --- | --- |
| G1 | 33 | - | - | 0.096 |
| G2 | 16 | G2 vs G1 | 0.122 |  |
| G3 | 15 | G3 vs G1 | **0.01** |  |


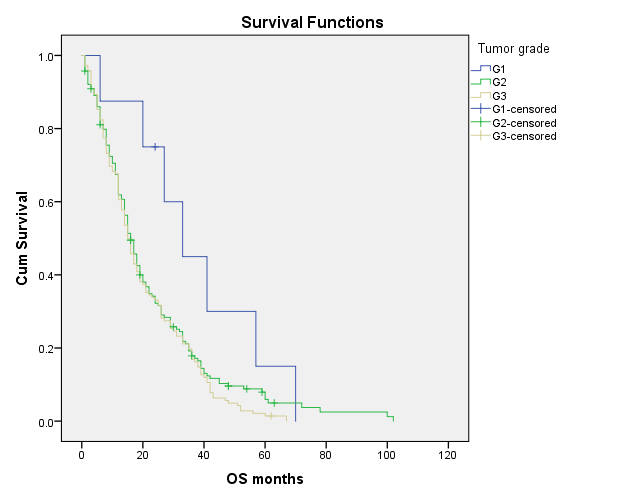


| No. at risk | | | | | | | |
| --- | --- | --- | --- | --- | --- | --- | --- |
| Months | 0 | 20 | 40 | 60 | 80 | 100 | 120 |
| G1 | 8 | 7 | 3 | 1 | 0 | 0 | 0 |
| G2 | 165 | 62 | 21 | 8 | 2 | 2 | 0 |
| G3 | 142 | 54 | 18 | 3 | 0 | 0 | 0 |

**Supplementary Figure S42. Kaplan–Meier estimates of OS according to grade status.**

OS, overall survival.

| Stage | Median OS (months) | Log-rank *P* value |
| --- | --- | --- |
| 3 | 16 | 0.764 |
| 4 | 17 |  |


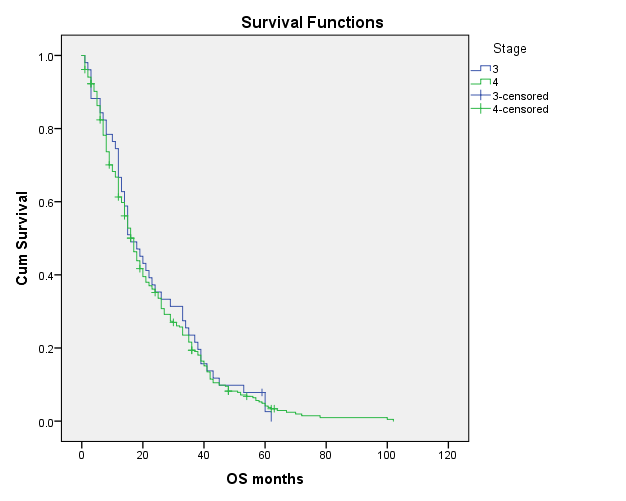


| No. at risk | | | | | | | |
| --- | --- | --- | --- | --- | --- | --- | --- |
| Months | 0 | 20 | 40 | 60 | 80 | 100 | 120 |
| 3 | 51 | 23 | 8 | 3 | 0 | 0 | 0 |
| 4 | 337 | 134 | 50 | 13 | 2 | 2 | 0 |

**Supplementary Figure S43. Kaplan–Meier estimates of OS according to stage.**

OS, overall survival.

| No. of metastasis site | Median OS (months) | Log-rank *P* value |
| --- | --- | --- |
| Multiple | 16 | 0.757 |
| Single | 17 |  |


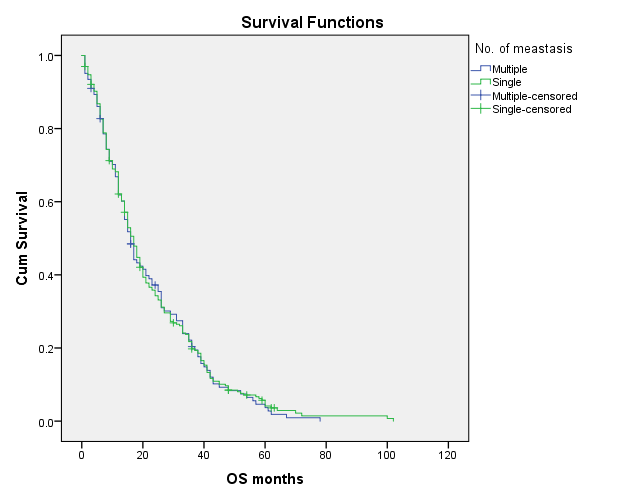


| No. at risk | | | | | | | |
| --- | --- | --- | --- | --- | --- | --- | --- |
| Months | 0 | 20 | 40 | 60 | 80 | 100 | 120 |
| Multiple | 122 | 49 | 17 | 5 | 0 | 0 | 0 |
| Single | 266 | 108 | 41 | 11 | 2 | 2 | 0 |

**Supplementary Figure S44. Kaplan–Meier estimates of OS according to no. of metastasis site (multiple vs single).**

OS, overall survival.

| No. of metastasis site | Median OS (months) | Log-rank *P* value |
| --- | --- | --- |
| >2 | 26 | 0.229 |
| ≤2 | 16 |  |


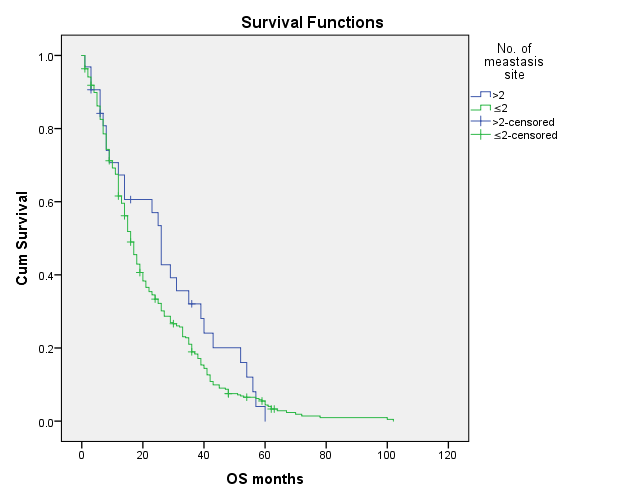


| No. at risk | | | | | | | |
| --- | --- | --- | --- | --- | --- | --- | --- |
| Months | 0 | 20 | 40 | 60 | 80 | 100 | 120 |
| >2 | 32 | 17 | 7 | 1 | 0 | 0 | 0 |
| ≤2 | 356 | 140 | 51 | 15 | 2 | 2 | 0 |

**Supplementary Figure S45. Kaplan–Meier estimates of OS according to no. of metastasis site (>2 vs ≤2).**

OS, overall survival.

| Site of metastases | Median OS (months) | Log-rank *P* value |
| --- | --- | --- |
| Bone | 19 | 0.279 |
| Other | 15 |  |


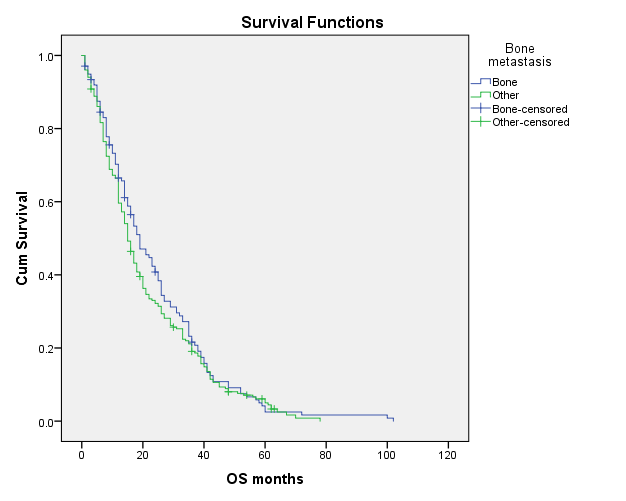


| No. at risk | | | | | | | |
| --- | --- | --- | --- | --- | --- | --- | --- |
| Months | 0 | 20 | 40 | 60 | 80 | 100 | 120 |
| Bone | 137 | 60 | 21 | 5 | 2 | 2 | 0 |
| Other | 251 | 97 | 37 | 11 | 0 | 0 | 0 |

**Supplementary Figure S46. Kaplan–Meier estimates of OS according to bone metastasis.**

OS, overall survival.

| Site of metastases | Median OS (months) | Log-rank *P* value |
| --- | --- | --- |
| Lung | 17 | 0.682 |
| Other | 16 |  |


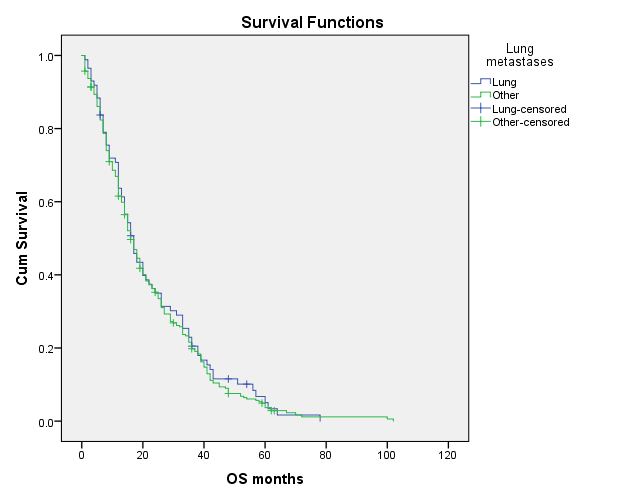


| No. at risk | | | | | | | |
| --- | --- | --- | --- | --- | --- | --- | --- |
| Months | 0 | 20 | 40 | 60 | 80 | 100 | 120 |
| Lung | 86 | 36 | 13 | 4 | 0 | 0 | 0 |
| Other | 302 | 121 | 45 | 12 | 2 | 2 | 0 |

**Supplementary Figure S47. Kaplan–Meier estimates of OS according to lung metastasis.**

OS, overall survival.

| Site of metastases | Median OS (months) | Log-rank *P* value |
| --- | --- | --- |
| Local | 18 | 0.778 |
| Other | 16 |  |


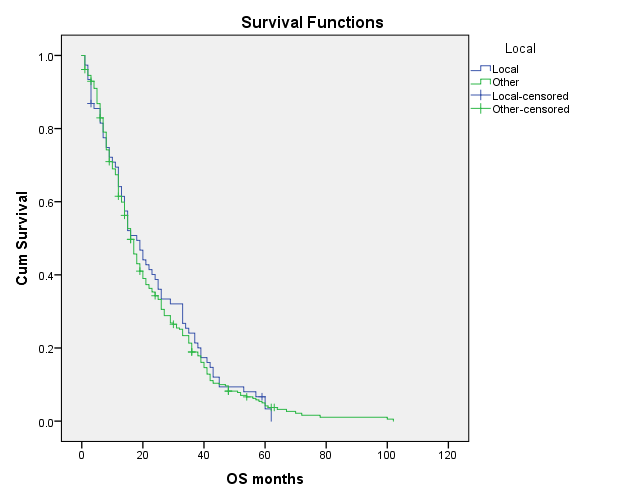


| No. at risk | | | | | | | |
| --- | --- | --- | --- | --- | --- | --- | --- |
| Months | 0 | 20 | 40 | 60 | 80 | 100 | 120 |
| Local | 76 | 35 | 13 | 4 | 0 | 0 | 0 |
| Other | 312 | 122 | 45 | 12 | 2 | 2 | 0 |

**Supplementary Figure S48. Kaplan–Meier estimates of OS according to local unresectable disease.**

OS, overall survival.

| Site of metastases | Median OS (months) | Log-rank *P* value |
| --- | --- | --- |
| Node | 15 | 0.878 |
| Other | 17 |  |


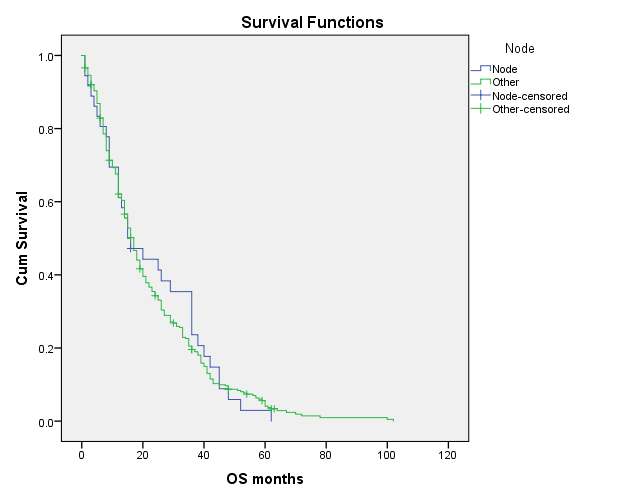


| No. at risk | | | | | | | |
| --- | --- | --- | --- | --- | --- | --- | --- |
| Months | 0 | 20 | 40 | 60 | 80 | 100 | 120 |
| Node | 36 | 16 | 7 | 1 | 0 | 0 | 0 |
| Other | 352 | 141 | 51 | 15 | 2 | 2 | 0 |

**Supplementary Figure S49. Kaplan–Meier estimates of OS according to node metastasis.**

OS, overall survival.

| Site of metastases | Median OS (months) | Log-rank *P* value |
| --- | --- | --- |
| Other | 17 | 0.766 |
| Pleural | 15 |  |


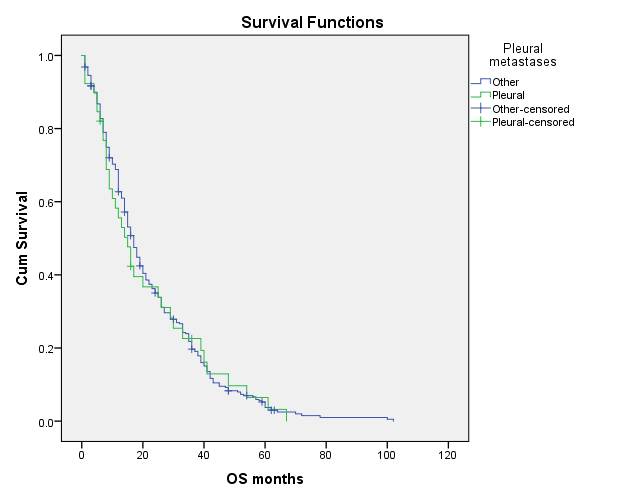


| No. at risk | | | | | | | |
| --- | --- | --- | --- | --- | --- | --- | --- |
| Months | 0 | 20 | 40 | 60 | 80 | 100 | 120 |
| Other | 349 | 143 | 52 | 14 | 2 | 2 | 0 |
| Pleural | 39 | 14 | 6 | 2 | 0 | 0 | 0 |

**Supplementary Figure S50. Kaplan–Meier estimates of OS according to pleural metastasis.**

OS, overall survival.

| Site of metastases | Median OS (months) | Log-rank *P* value |
| --- | --- | --- |
| Other | 16 | 0.084 |
| Skin | 21 |  |


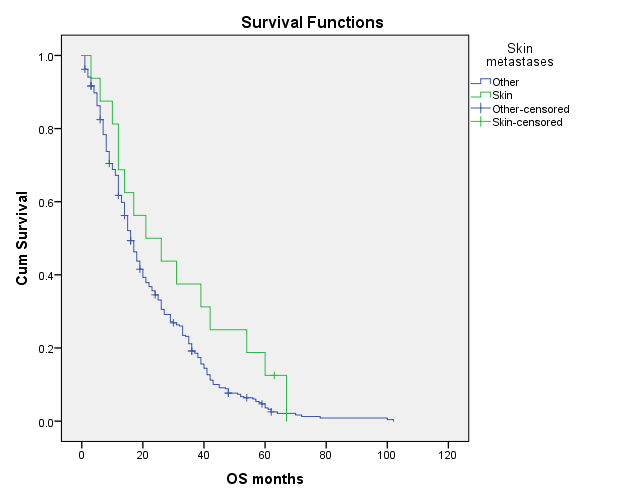


| No. at risk | | | | | | | |
| --- | --- | --- | --- | --- | --- | --- | --- |
| Months | 0 | 20 | 40 | 60 | 80 | 100 | 120 |
| Other | 372 | 148 | 53 | 13 | 2 | 2 | 0 |
| Skin | 16 | 9 | 5 | 3 | 0 | 0 | 0 |

**Supplementary Figure S51. Kaplan–Meier estimates of OS according to skin metastasis.**

OS, overall survival.

| Site of metastases | Median OS (months) | Log-rank *P* value |
| --- | --- | --- |
| Other | 16 | 0.281 |
| Other B. | 22 |  |


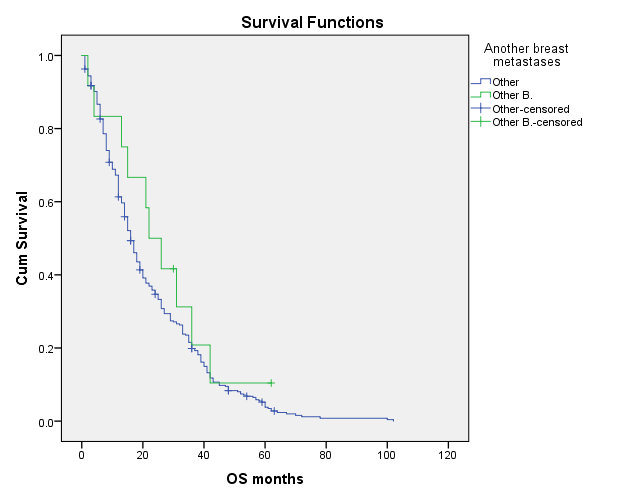


| No. at risk | | | | | | | |
| --- | --- | --- | --- | --- | --- | --- | --- |
| Months | 0 | 20 | 40 | 60 | 80 | 100 | 120 |
| Other | 376 | 149 | 56 | 15 | 2 | 2 | 0 |
| Other B. | 12 | 8 | 2 | 1 | 0 | 0 | 0 |

**Supplementary Figure S52. Kaplan–Meier estimates of OS according to the other breast metastasis.**

B, breast; OS, overall survival.

| Site of metastases | Median OS (months) | Log-rank *P* value |
| --- | --- | --- |
| Ascites | 6 | 0.428 |
| Other | 16 |  |


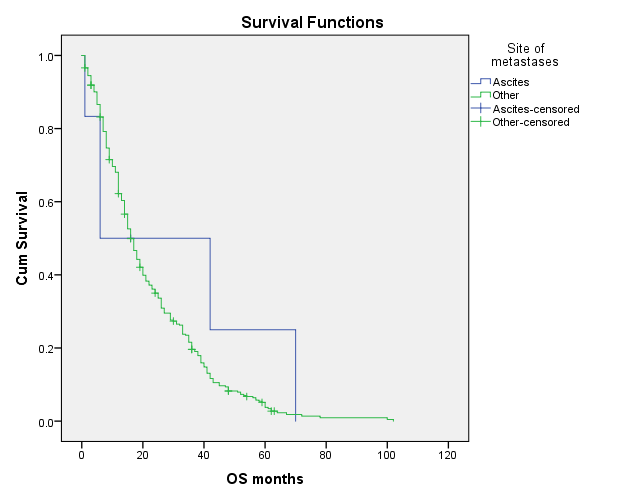


| No. at risk | | | | | | | |
| --- | --- | --- | --- | --- | --- | --- | --- |
| Months | 0 | 20 | 40 | 60 | 80 | 100 | 120 |
| Ascites | 6 | 2 | 2 | 1 | 0 | 0 | 0 |
| Other | 382 | 155 | 56 | 15 | 2 | 2 | 8 |

**Supplementary Figure S53. Kaplan–Meier estimates of OS according to ascites.**

OS, overall survival.

| Site of metastases | Median OS (months) | Log-rank *P* value |
| --- | --- | --- |
| Other | 16 | 0.758 |
| Pericar. | 17 |  |


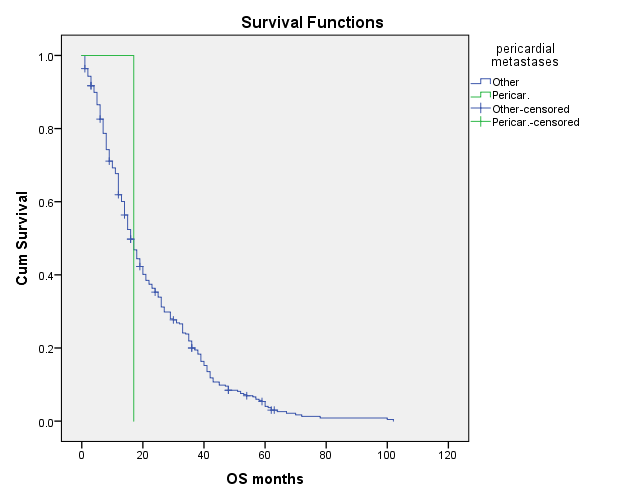


| No. at risk | | | | | | | |
| --- | --- | --- | --- | --- | --- | --- | --- |
| Months | 0 | 20 | 40 | 60 | 80 | 100 | 120 |
| Other | 387 | 157 | 58 | 16 | 2 | 2 | 0 |
| Pericar. | 1 | 0 | 0 | 0 | 0 | 0 | 0 |

**Supplementary Figure S54. Kaplan–Meier estimates of OS according to pericardial metastasis.**

OS, overall survival; Pericar, pericardial.

| Site of metastases | Median OS (months) | Log-rank *P* value |
| --- | --- | --- |
| Other | 17 | 0.238 |
| Spleen | 9 |  |


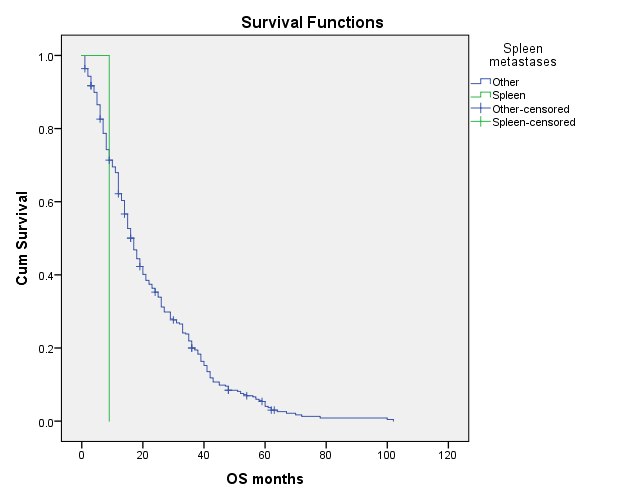


| No. at risk | | | | | | | |
| --- | --- | --- | --- | --- | --- | --- | --- |
| Months | 0 | 20 | 40 | 60 | 80 | 100 | 120 |
| Other | 387 | 157 | 58 | 16 | 2 | 2 | 0 |
| Spleen | 1 | 0 | 0 | 0 | 0 | 0 | 0 |

**Supplementary Figure S55. Kaplan–Meier estimates of OS according to spleen metastasis.**

OS, overall survival.

| CEA levels at diagnosis | Median OS (months) | Log-rank *P* value |
| --- | --- | --- |
| Normal | 16 | 0.071 |
| High | 13 |  |


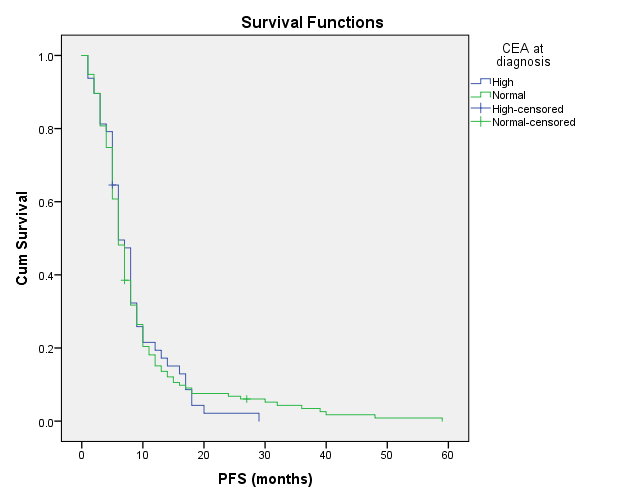


| No. at risk | | | | | | | |
| --- | --- | --- | --- | --- | --- | --- | --- |
| Months | 0 | 20 | 40 | 60 | 80 | 100 | 120 |
| Normal | 136 | 54 | 21 | 8 | 1 | 1 | 0 |
| High | 50 | 13 | 4 | 2 | 0 | 0 | 0 |

**Supplementary Figure S56. Kaplan–Meier estimates of OS according to CEA levels** **at diagnosis.**

CEA, carcinoembryonic antigen; OS, overall survival.

| CA15-3 levels at diagnosis | Median OS (months) | Log-rank *P* value |
| --- | --- | --- |
| Normal | 15 | 0.721 |
| High | 14 |  |


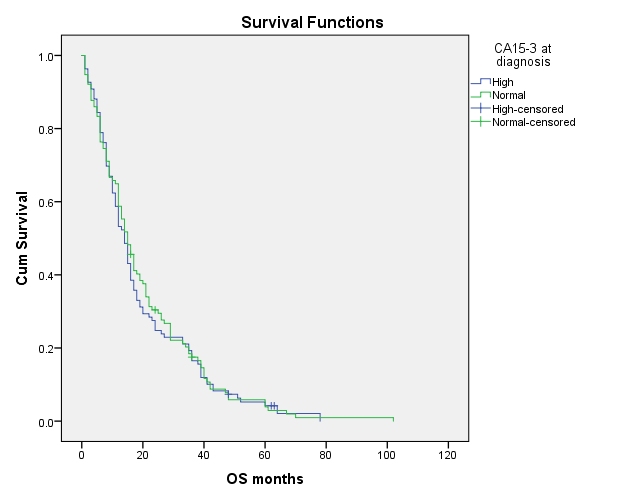


| No. at risk | | | | | | | |
| --- | --- | --- | --- | --- | --- | --- | --- |
| Months | 0 | 20 | 40 | 60 | 80 | 100 | 120 |
| Normal | 114 | 43 | 15 | 6 | 1 | 1 | 0 |
| High | 109 | 34 | 13 | 5 | 0 | 0 | 0 |

**Supplementary Figure S57. Kaplan–Meier estimates of OS according to CA15-3 levels** **at diagnosis.**

CA15-3, cancer antigen 15-3; OS, overall survival.

| Treatment choice | Median OS (months) |  | Log-rank *P* value | Log-rank *P* value (overall) |
| --- | --- | --- | --- | --- |
| Anti-HER2 | 22 | Anti-HER2 vs ET | 0.647 | 0.583 |
|  |  | Anti-HER2 vs Chemotherapy | 0.328 |  |
| Chemotherapy | 16 | Chemotherapy vs ET | 0.736 |  |
| ET | 25 | - | - |  |


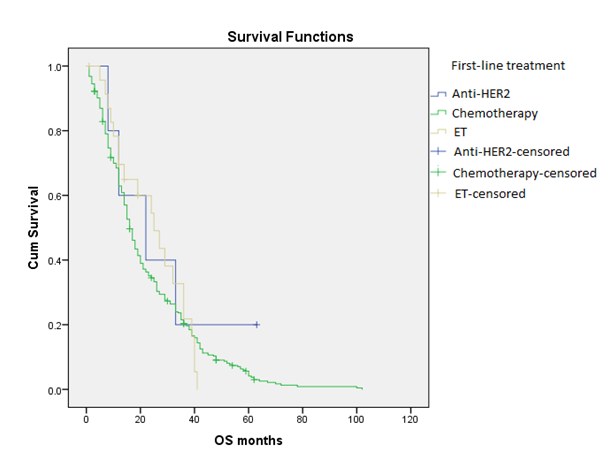


| No. at risk | | | | | | | |
| --- | --- | --- | --- | --- | --- | --- | --- |
| Months | 0 | 20 | 40 | 60 | 80 | 100 | 120 |
| Anti-HER2 | 5 | 3 | 1 | 1 | 0 | 0 | 0 |
| Chemotherapy | 345 | 139 | 53 | 15 | 2 | 2 | 0 |
| ET | 24 | 11 | 3 | 0 | 0 | 0 | 0 |

**Supplementary Figure S58. Kaplan–Meier estimates of OS according to first line treatment (ET vs anti-HER2 vs Chemotherapy).**

HER2, human epidermal growth factor receptor 2; ET, endocrine therapy; OS, overall survival.

| HR-positive /HER2-negative | Median OS (months) | Log-rank *P* value |
| --- | --- | --- |
| Chemotherapy | 17 | 0.855 |
| ET | 27 |  |


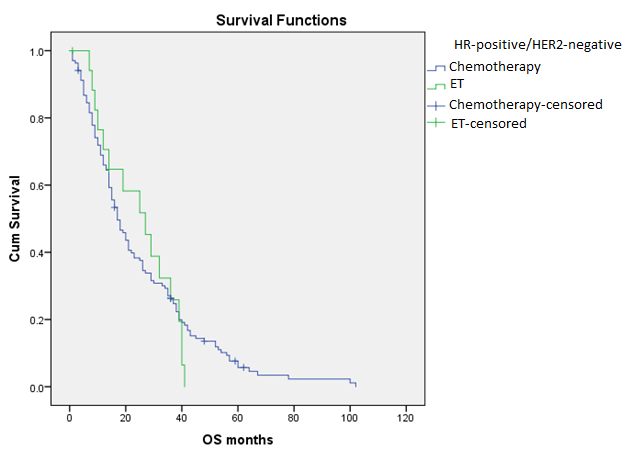


| No. at risk | | | | | | | |
| --- | --- | --- | --- | --- | --- | --- | --- |
| Months | 0 | 20 | 40 | 60 | 80 | 100 | 120 |
| Chemotherapy | 136 | 61 | 25 | 8 | 2 | 2 | 0 |
| ET | 18 | 9 | 3 | 0 | 0 | 0 | 0 |

**Supplementary Figure S59. Kaplan–Meier estimates of OS according to first-line treatment in patients with HR-positive/HER2-negative (ET vs chemotherapy).**

HER2, human epidermal growth factor receptor 2; HR, hormonal receptor; ET, endocrine therapy; OS, overall survival.

| First-line treatment | Median OS (months) | Log-rank *P* value (overall) |
| --- | --- | --- |
| Anthracycline-based | 26 | **0.003** |
| Anti-HER2 | 22 |  |
| AT | 20 |  |
| ET | 25 |  |
| Other | 14 |  |
| TXN-based | 18 |  |


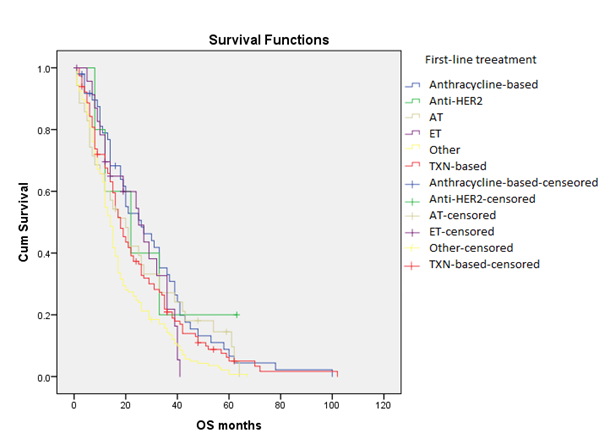


| No. at risk | | | | | | | |
| --- | --- | --- | --- | --- | --- | --- | --- |
| Months | 0 | 20 | 40 | 60 | 80 | 100 | 120 |
| Anthracycline-based | 49 | 28 | 12 | 4 | 1 | 1 | 0 |
| Anti-HER2 | 5 | 3 | 1 | 1 | 0 | 0 | 0 |
| AT | 35 | 17 | 8 | 3 | 0 | 0 | 0 |
| ET | 24 | 11 | 3 | 0 | 0 | 0 | 0 |
| Other | 124 | 43 | 15 | 3 | 0 | 0 | 0 |
| TXN-based | 115 | 51 | 18 | 5 | 1 | 1 | 0 |

**Supplementary Figure S60. Kaplan–Meier estimates of OS according to first-line treatment.**

*Others include: capecitabine (single agent), cyclophosphamide + methotrexate + fluorouracil, gemcitabine + vinorelbine, platinum + gemcitabine, platinum + vinorelbine, vinorelbine (single agent), vinorelbine + capecitabine.

AT, doxorubicin and docetaxel; HER2, human epidermal growth factor receptor 2; ET, endocrine therapy; OS, overall survival; TXN, taxane.
